# Supplementary material for: Flexible supercapacitor electrodes based on real metal-like cellulose papers
Source: Nat Commun. 2017 Sep 14;8:536. doi: 10.1038/s41467-017-00550-3 (PMC5599591; doi:10.1038/s41467-017-00550-3)
Supplement: Supplementary file 1 — Supplementary Information [file 41467_2017_550_MOESM1_ESM.pdf]

Description of Supplementary Files

File Name: Supplementary Information
Description: Supplementary figures, Supplementary Tables, Supplementary References

File Name: Peer Review File

Supplementary Information

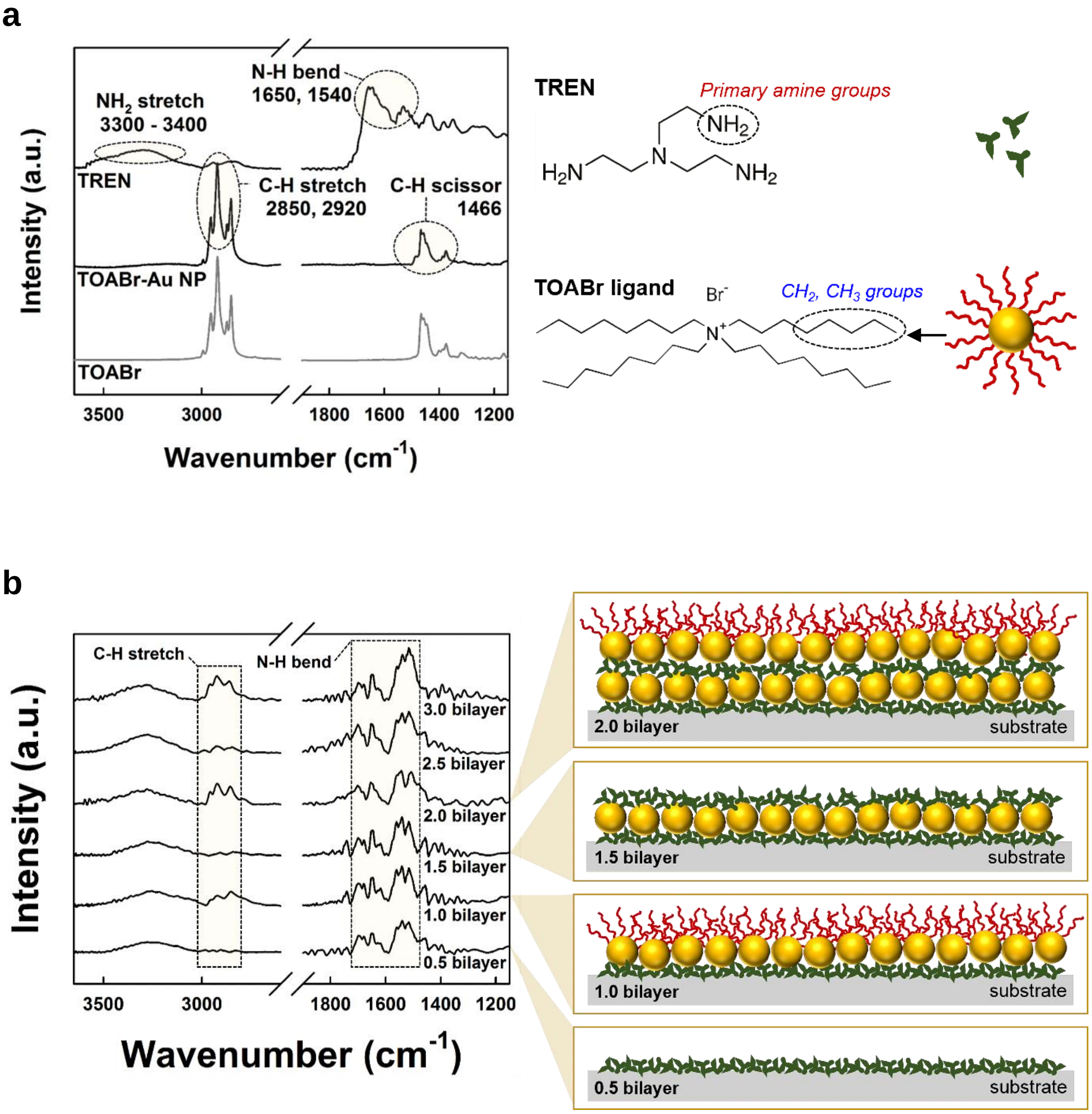

Supplementary Figure 1. LbL growth mechanism of (TREN/TOABr-Au NP)$_n$ multilayers. a, Characteristic FTIR spectra and the molecular structures of TREN, TOABr-Au NPs, and native TOABr ligands. **b,** FTIR spectra and scheme of molecular ligand-mediated LbL-assembled (TREN/TOABr-Au NP)$_n$ multilayers as a function of the bilayer

number (n). As shown in the FTIR spectra (Supplementary Fig. 1b), the deposition of TREN onto the TOABr-Au NP (with a diameter of ~ 8 nm)-coated substrates gradually eliminated the ammonium group-functionalized TOABr ligands (see the C-H stretching peaks at 2,850 − 2,950 cm^{-1}) loosely bound to the surface of the Au NPs because the primary amine moieties of TREN (see the $-NH_2$ stretching peaks at 3,300 − 3,400 cm^{-1} and N-H bending peaks at 1,540 − 1,650 cm^{-1}) as an electron donor had a higher affinity (by covalent bonding) for the surface of the various metal or metal oxide NPs and Au NPs, than for functional groups such as the ammonium groups of the TOABr ligands or carboxylate ion (COO^{-}) groups of the oleic acid (OA) ligands. Therefore, the alternating deposition of TREN and TOABr-Au NPs repeated the generation and disappearance of the C-H stretching peaks (at 2,850 − 2,950 cm^{-1}) originating from the TOABr ligands when the outermost layer was changed from TREN to TOABr-Au NPs and vice versa.

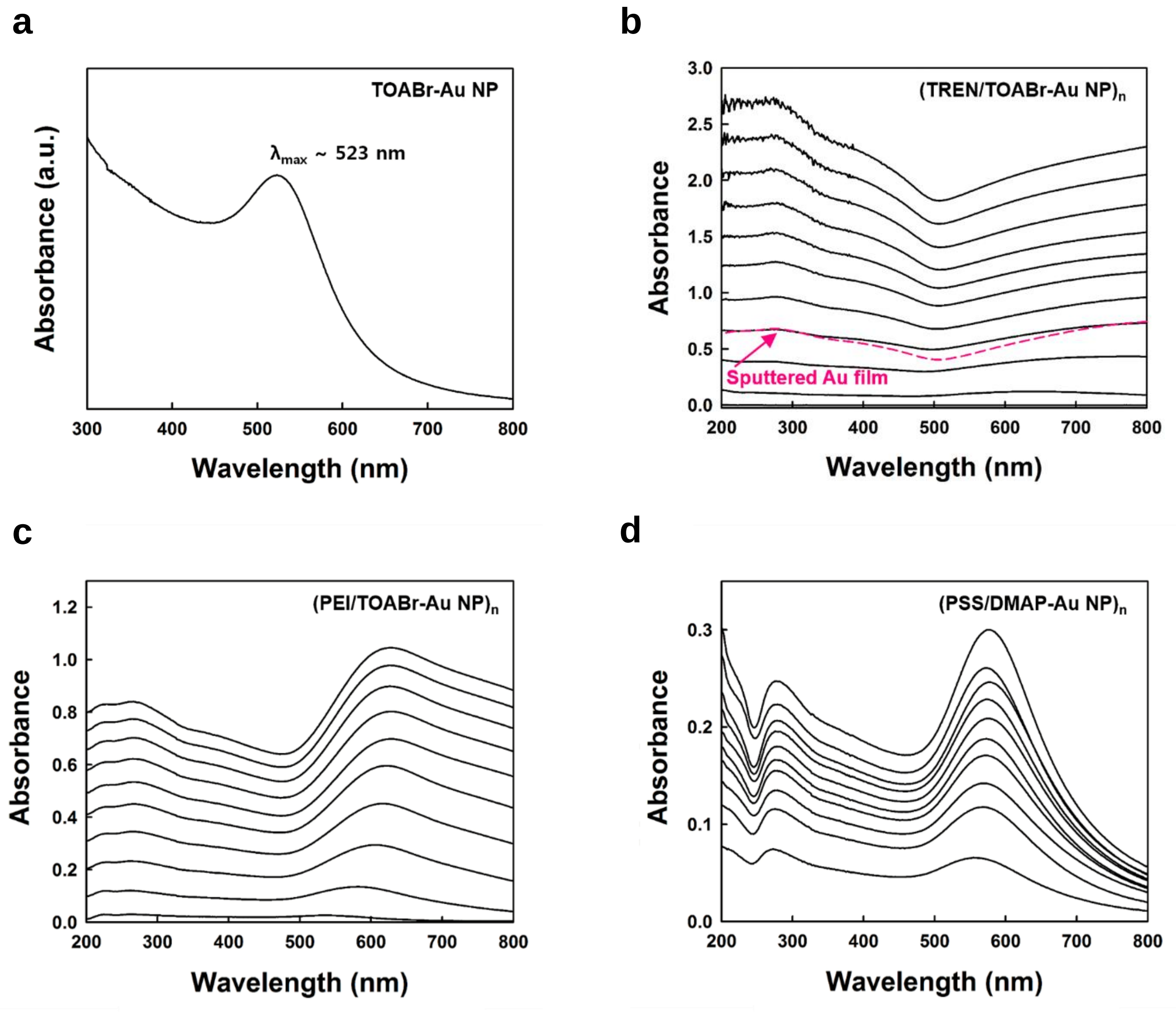

Supplementary Figure 2. UV-vis spectra of Au NP-based multilayers. UV-Vis spectra of the **a,** TOABr-Au NP, **b,** $(TREN/TOABr\text{-}Au\ NP)_n$, **c,** $(PEI/TOABr\text{-}Au\ NP)_n$, and **d,** $(PSS/DMAP\text{-}Au\ NP)_n$ multilayers as a function of the bilayer number (*n*). Au NP multilayers mediated by small molecule ligands displayed a dramatic change in the surface plasmon absorbance spectra with the increasing bilayer number. As shown in Supplementary Fig. 2b, the UV-Vis absorption spectra of the TOABr-Au NPs with a surface plasmon absorption peak (λ_{max}) at 523 nm in toluene solution (Supplementary Fig. 2a) were strongly redshifted to the near-infrared (IR) region; the surface plasmon absorption peak disappeared as the bilayer number of the $(TREN/TOABr\text{-}Au\ NP)_n$ multilayer increased only up to 3 bilayers. We also confirmed that the UV-Vis spectra of the multilayers were similar to the spectra of the

sputtered 50 nm thick Au films (red dashed line in Supplementary Fig. 2b). These results suggest that the interparticle distance among the Au NPs significantly decreased in both the lateral and vertical dimensions. Additionally, these Au NP-based films can be used for various plasmonic applications, including near-IR absorbing materials. However, in the case of using the amine-functionalized polymers (i.e., PEI) with the ligand exchange reaction instead of TREN (i.e., the $(PEI/TOABr\text{-}Au\ NP)_n$ multilayers)), the plasmon absorption peak of the Au NPs in the film with 10 bilayers was redshifted to approximately 610 nm, accompanied by broadening of the peak (Supplementary Fig. 2c). Not surprisingly, the electrostatic LbL-assembled films composed of cationic 4-dimethylaminopyridine (DMAP)-stabilized Au NPs (4-dimethylaminopyridine, DMAP) and anionic poly(4-styrenesulfonic acid) (PSS) (i.e., $(PSS/DMAP\text{-}Au\ NP)_n$ multilayers) also showed a slight peak shift and peak broadening with relatively low absorbance intensity (Supplementary Fig. 2d).

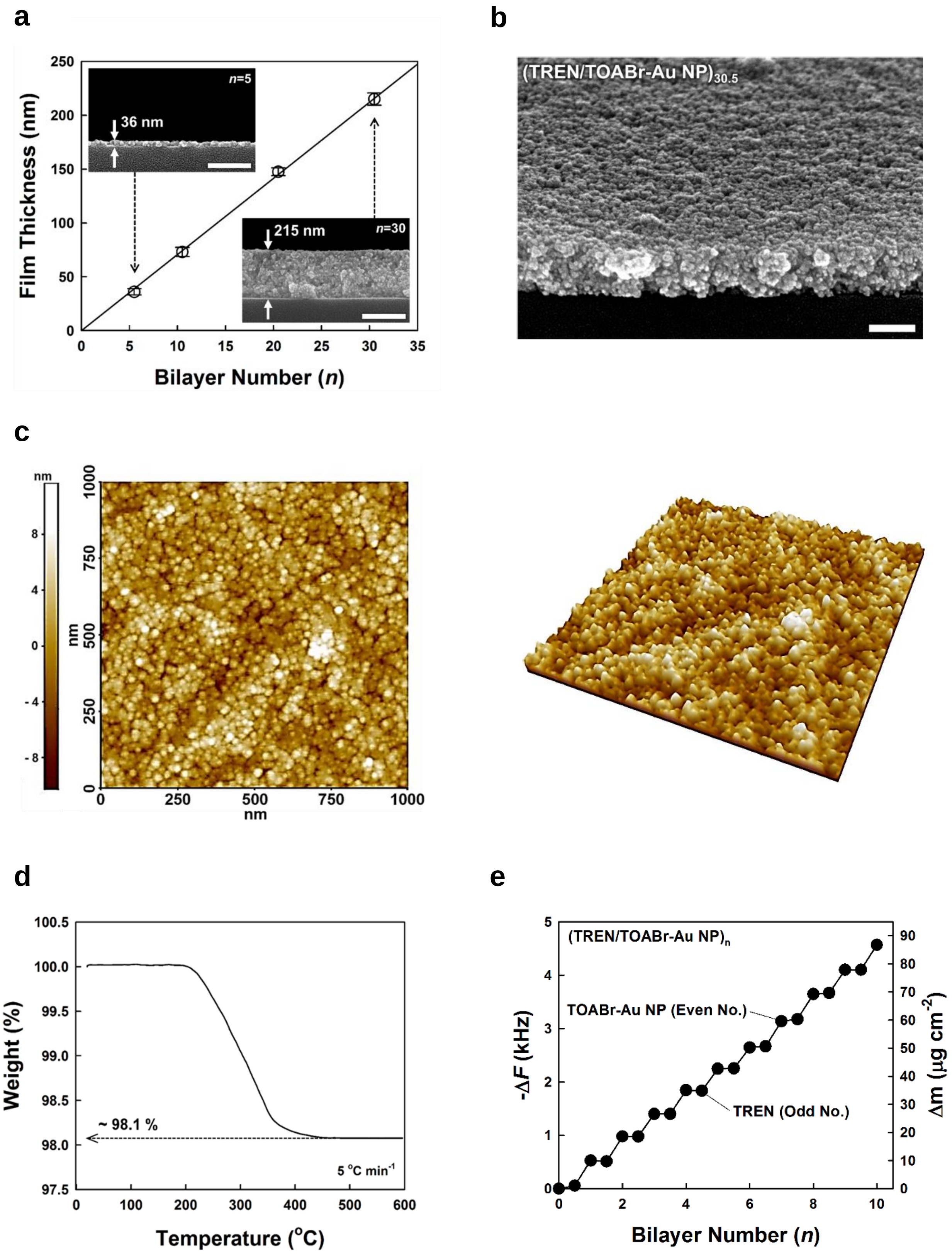
a
Film Thickness (nm)
Bilayer Number (n)
n=5
36 nm
n=30
215 nm
b
(TREN/TOABr-Au NP)30.5
c
nm
d
Weight (%)
Temperature (°C)
~ 98.1 %
5 °C min-1
e
(TREN/TOABr-Au NP)n
TOABr-Au NP (Even No.)
TREN (Odd No.)
-ΔF (kHz)
Δm (μg cm-2)
Bilayer Number (n)

Supplementary Figure 3. Physical properties of (TREN/TOABr-Au NP)$_n$ multilayers. a, Thickness of (TREN/TOABr-Au NP)$_n$ multilayer films as a function of the bilayer number (*n*). Error bars show the standard deviation of the thickness obtained from three samples. **b,** Tilted SEM and **c,** AFM topographic images of (TREN/TOABr-Au NP)$_{30.5}$ multilayers deposited onto the silicon wafer substrate. **d,** TGA data, and **e,** QCM data for the (TREN/TOABr-Au NP)$_n$ multilayer films. All scale bars indicate 200 nm. The film thickness of the formed multilayer was monitored using cross-sectional FE-SEM. In this case, the total thickness of the (TREN/TOABr-Au NP)$_n$ multilayer films was regularly increased from 36 (for n = 5.5) to 219 nm (for n = 30.5), implying that the average thickness per bilayer was approximately 7.3 nm (Supplementary Fig. 3a). Additionally, the formed (TREN/TOABr-Au NP)$_{30.5}$ multilayers displayed the dense and disordered structure with nanopores (Supplementary Figs. 3b and 3c). In this case, the root-mean-square (RMS) roughness of the film with 30 bilayers was measured to be approximately 2.65 nm within a scan area of 1 µm × 1 µm. Additionally, given that the mass ratio of the Au NPs within the multilayers (by TGA) and the total loading amount of the film with 10 bilayers (by QCM) was measured to be approximately 98.1 %, and 86.7 $\mu g \cdot cm^{-2}$, respectively (Supplementary Figs. 3d and 3e). As a result, the small organic molecule-mediated LbL assembly in organic media favored Au NPs with densely packed arrays in the lateral and vertical dimension, remarkably reducing the NP-NP distance.

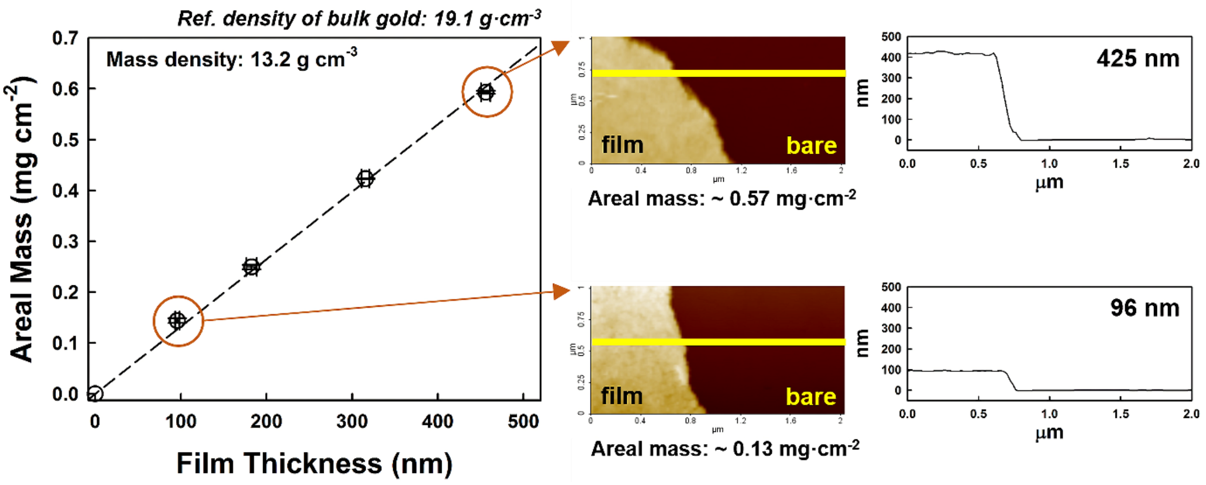

Supplementary Figure 4. Mass density of (TREN/TOABr-Au NP)$_n$ multilayers. A plot of areal mass (mg·cm^{-2}) vs. film thickness (nm) of (TREN/TOABr-Au NP)$_n$ multilayers deposited on QCM electrodes (left) and thickness profiles at two different points measured using AFM (right). In this case, the (TREN/TOABr-Au NP)$_{30.5}$ multilayers had a high mass density of 13.2 g·cm^{-3} (the mass density of bulk gold: ~ 19.1 g·cm^{-3}), a porosity of 30.8 %, and a pore volume of 0.022 cm^3·g^{-1}, respectively. The porosity of the multilayer film was calculated by the following equation: porosity (%) = (1 – (ρ_{film}/ρ_{bulk})) × 100, where ρ is the mass density. Error bars show the standard deviation of the each values obtained from three samples.

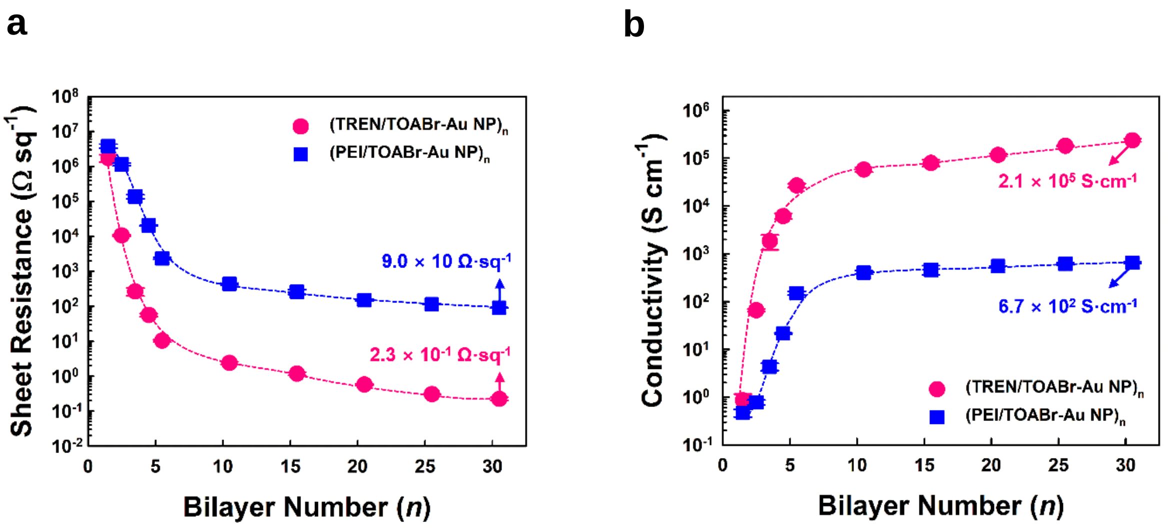

Supplementary Figure 5. Electrical properties of Au-based multilayers. a, Sheet resistance and **b,** conductivity change of (TREN/TOABr-Au NP)$_n$ and (PEI/TOABr-Au NP)$_n$ multilayers as a function of the bilayer number (*n*), respectively.

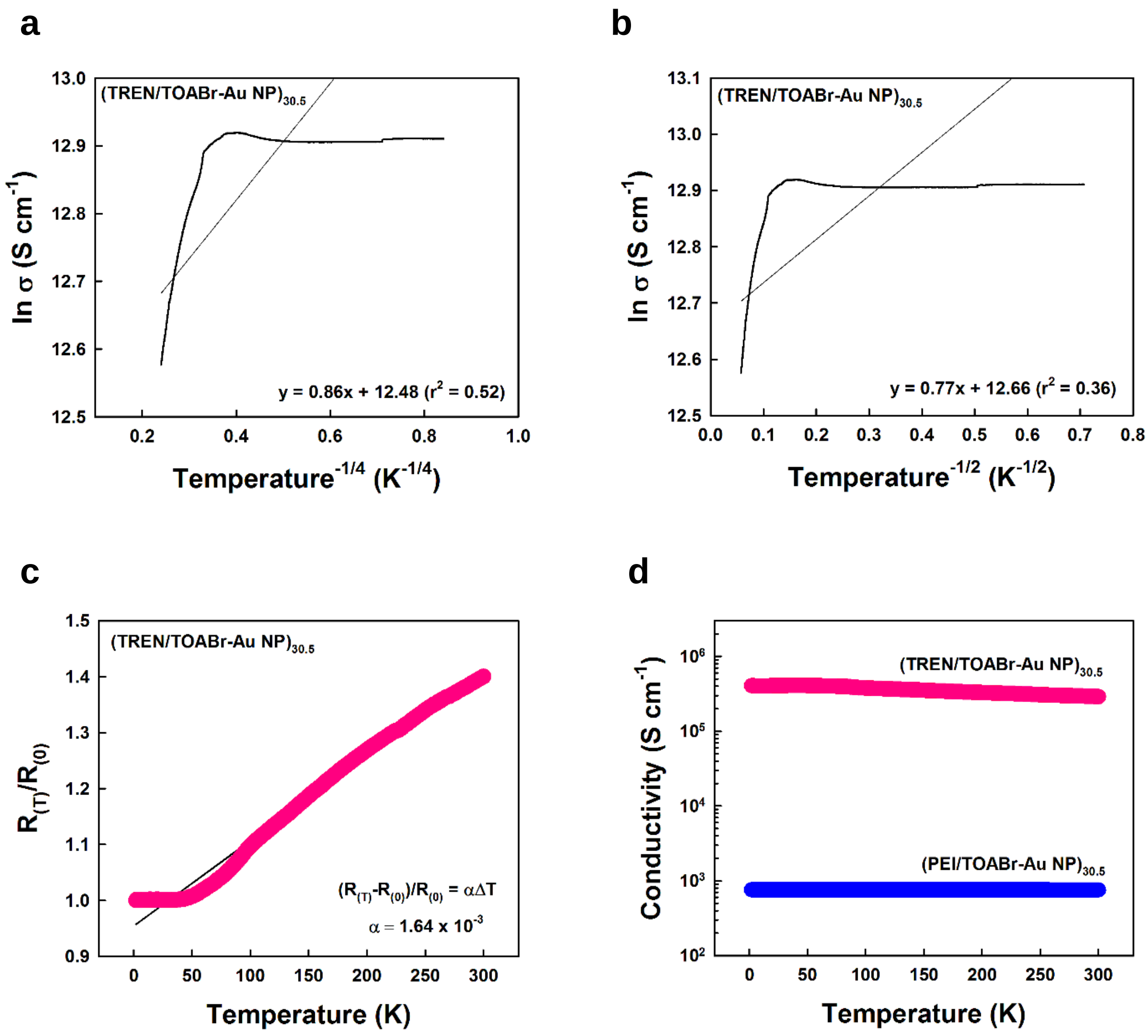

Supplementary Figure 6. Charge transport mechanism of the Au NP-based multilayers. **a,** Plots of *ln*σ *vs* $T^{-1/4}$ for the hopping mechanism and **b,** plots of *ln*σ *vs* $T^{-1/2}$ for the tunneling mechanism of the (TREN/TOABr-Au NP)$_{30.5}$ multilayer films. **c,** Resistance ($R_{(T)}/R_{(0)}$) vs. temperature (K) for the (TREN/TOABr-Au NP)$_{30.5}$ multilayer films. **d,** Temperature dependence of the conductivity for the (TREN/TOABr-Au NP)$_{30.5}$ and (PEI/TOABr-Au NP)$_{30.5}$ multilayer films. The electron transport mechanism for semiconductors can be demonstrated by the various-range hopping (VRH) and tunneling conduction processes as follows: $\sigma = \sigma_0\, exp(-A/T^{(1/d+1)})$, where σ is the conductivity, T is the absolute temperature (K), A is a constant, and d is the dimensionality. As shown in

Supplementary Figs. 6a and 6b, the conductivity ($ln\ \sigma$) as a function of $T^{-1/4}$ (for hopping) or $T^{-1/2}$ (for tunneling) for the (TREN/TOABr-Au NP)$_{30.5}$ multilayers does not correspond to a linear dependence, suggesting metallic conduction between the adjacent Au NPs. In Supplementary Fig. 6c, The (TREN/TOABr-Au NP)$_{30.5}$ multilayers also exhibited a positive temperature coefficient of 1.64×10^{-3} K^{-1}, which is characteristic of pure metals, obtained by the following relationship: $\Delta R/R_0 = \alpha \Delta T$, where R and α are the resistance (Ω) and the temperature coefficient, respectively. The electric conductivity of the (TREN/TOABr-Au NP)$_{30.5}$ multilayer films was higher than that of the (PEI/TOABr-Au NP)$_{30.5}$ multilayers, and it was maintained within the entire temperature range (2 − 300 K) (Supplementary Fig. 6d).

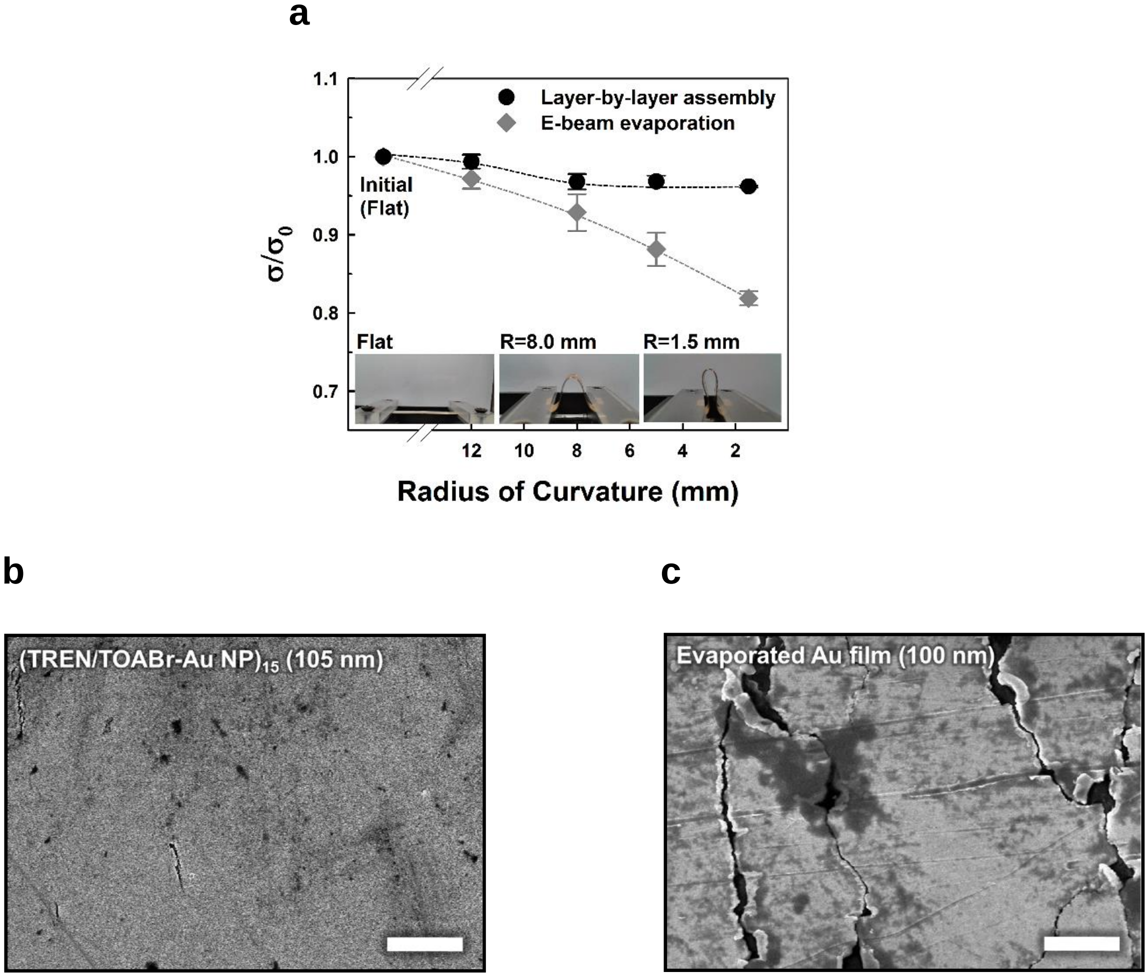

Supplementary Figure 7. Mechanical properties of (TREN/TOABr-Au NP)$_n$ films. a, Electrical stability of 105 nm-thick (TREN/TOABr-Au NP)$_{15}$ multilayer-coated, and 100 nm-thick E-beam evaporated PET films as a function of the bending radius (i.e., 12, 8, 5, and 1.5 mm). FE-SEM images of **b,** LbL assembled (TREN/TOABr-Au NP)$_{15}$ multilayer film (105 nm) and **c,** Au evaporated film (100 nm) after 10,000 cycles. In this case, the evaporated Au-coated PET film generated the large cracks (white arrows) that cause the increase of internal resistance after bending test. All scale bars indicate 2 µm.

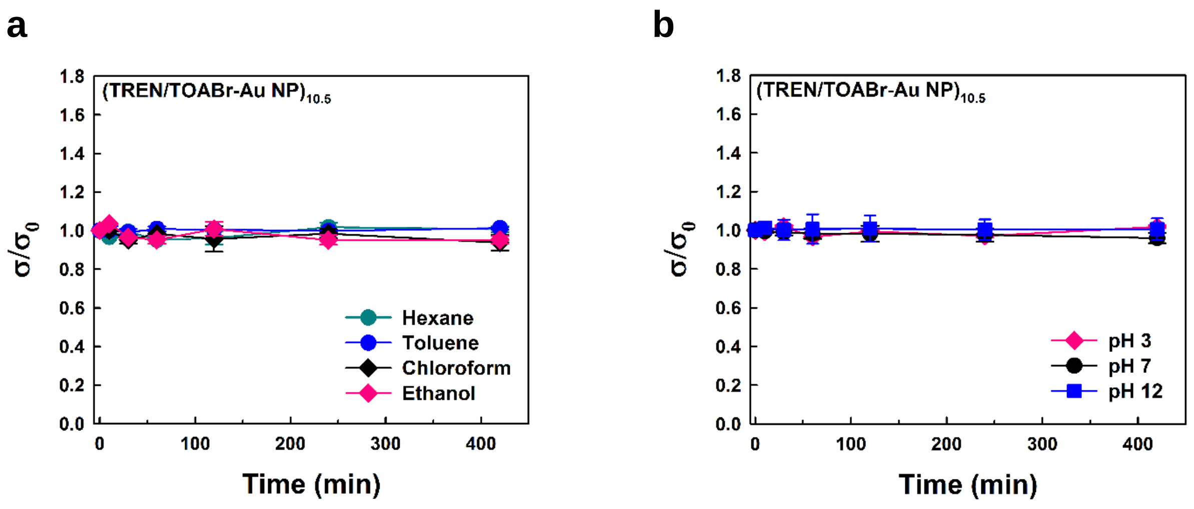

Supplementary Figure 8. Film stability in the various media. Change in electric conductivity ratio (σ/σ_0) measured for the (TREN/TOABr-Au NP)$_{10.5}$ multilayers as a function of **a,** immersion time in hexane, toluene, chloroform, and ethanol solvents, and **b,** immersion time in aqueous solutions of pH 3, 7, and 12. In this case, the electric conductivity of all the samples was measured after sufficient removal of residual solvent in vacuum.

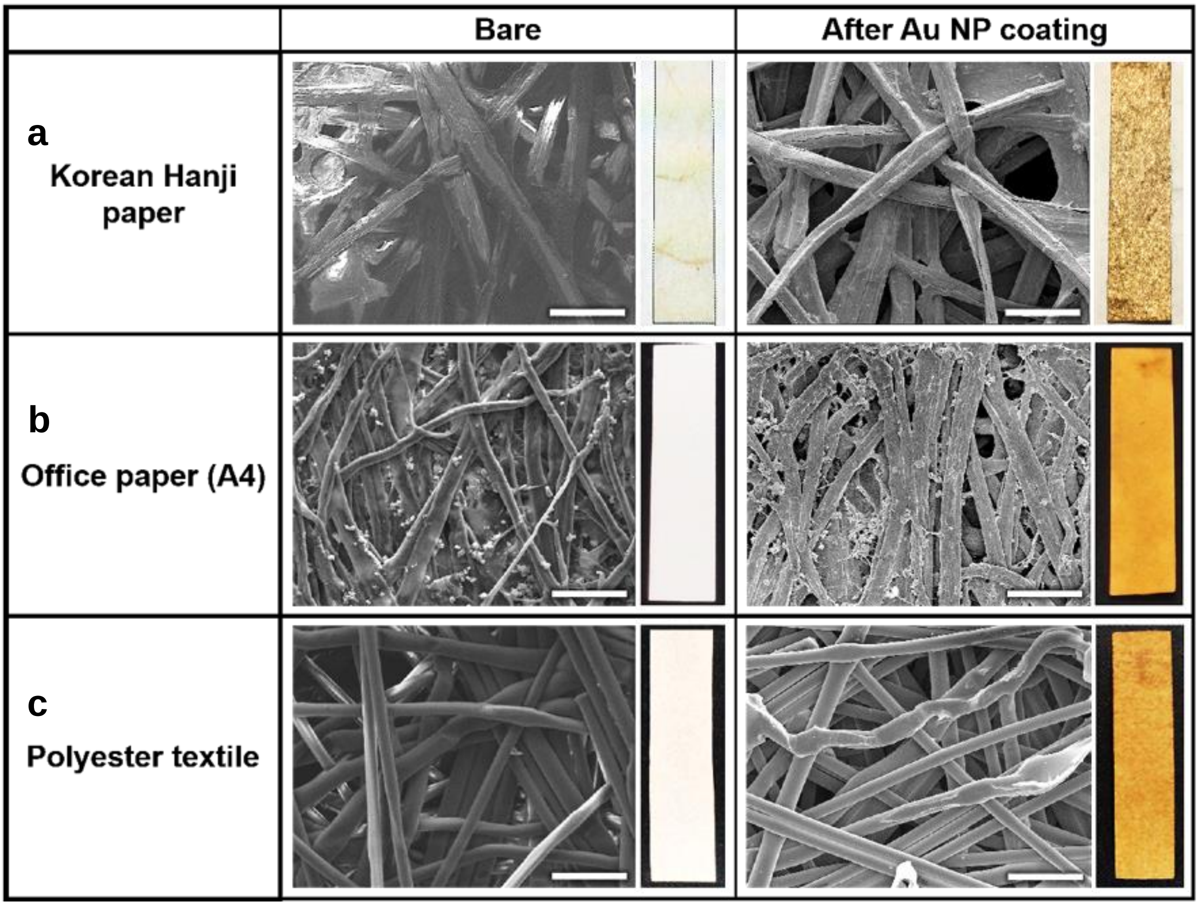

Supplementary Figure 9. FE-SEM and digital images of the various metallic textiles. a, Korean Hanji paper (cellulose paper), **b,** commercial office paper (A4 paper sheet), and **c,** polyester textile before and after deposition of (TREN/TOABr-Au NP)$_{10.5}$ multilayers. In this case, all images were obtained without the conductive coating. All scale bars in SEM images correspond to 50 µm.

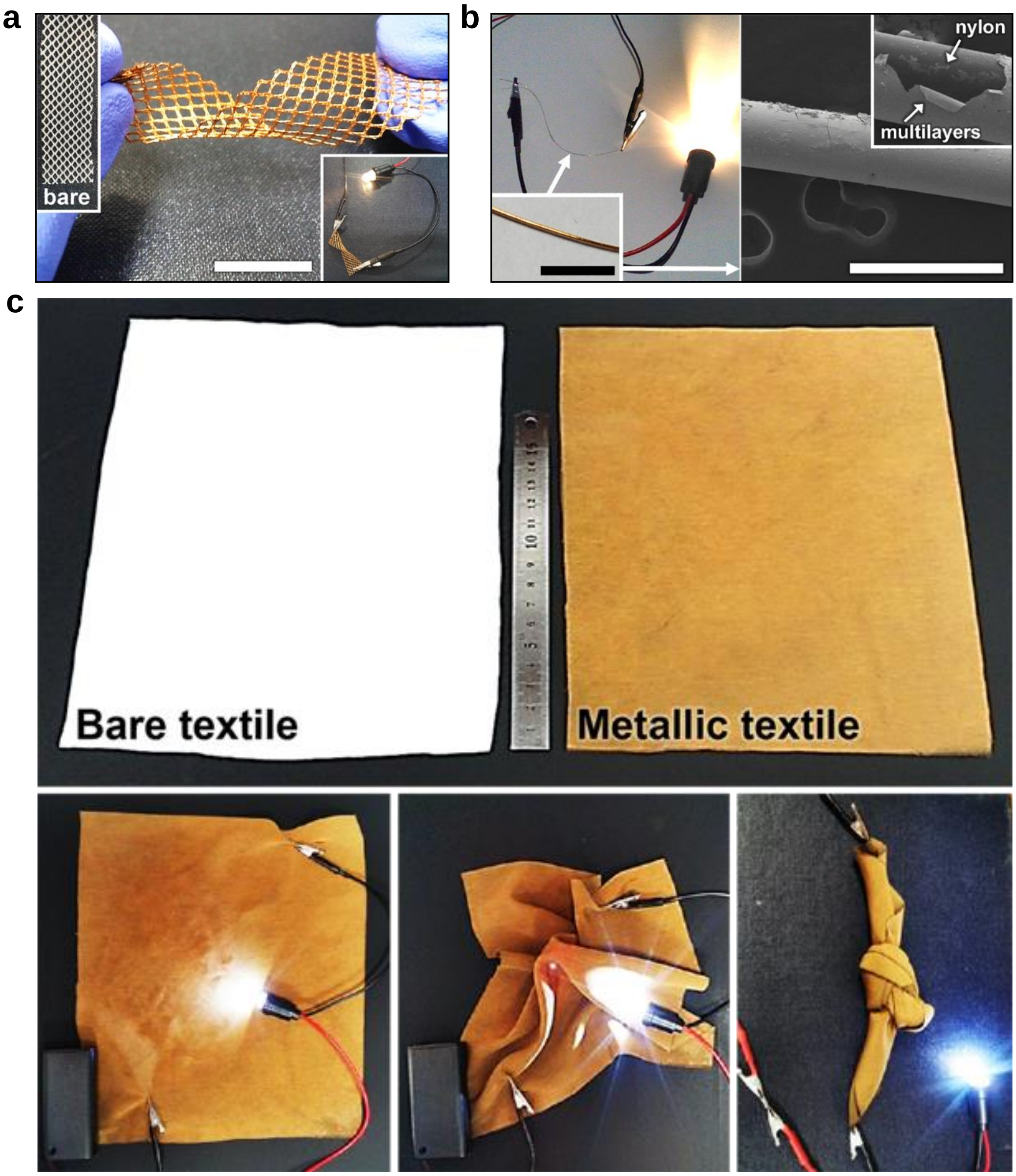

Supplementary Figure 10. Wide application to the various textile substrates. Photographs of (TREN/TOABr-Au NP)$_n$ multilayer-coated **a,** polyester mesh, **b,** nylon thread (ø ≈ 200 μm), and **c,** large area (20 cm × 22 cm) polyester cloth (top-left indicates bare polyester cloth). The right side of Supplementary Fig. 9b shows the FE-SEM images of (TREN/TOABr-Au NP)$_{20.5}$ multilayers-coated nylon thread. Additionally, the bottom images in **c** indicate a LED connection with normal (left), crumpled (middle), and knotted (right) polyester cloth. Scale bars **a** and **b** (including inset) indicate 1 cm and 500 μm, respectively.

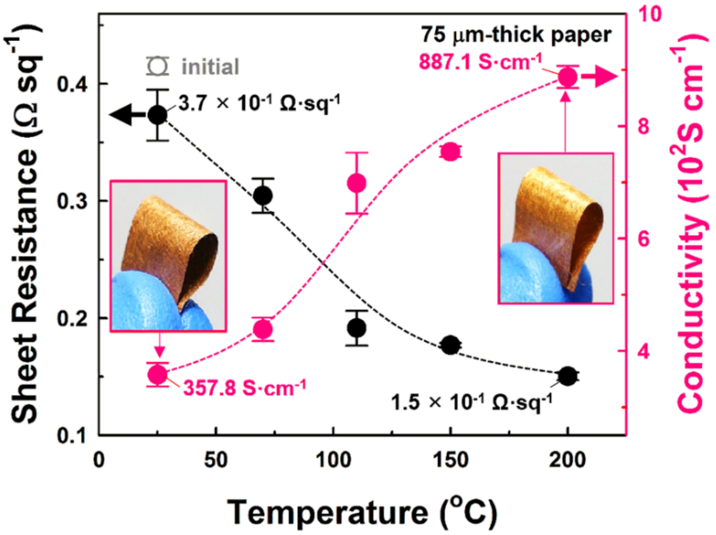

Supplementary Figure 11. Electrical properties of MPs. Sheet resistance and electric conductivity of 75 μm-thick (TREN/TOABr-Au NP)$_{10.5}$ multilayer-coated cellulose paper as a function of annealing temperature under mechanical pressing. The initial value (gray open circle) indicates the (TREN/TOABr-Au NP)$_{10.5}$ multilayer-coated cellulose paper without mechanical pressing or heating.

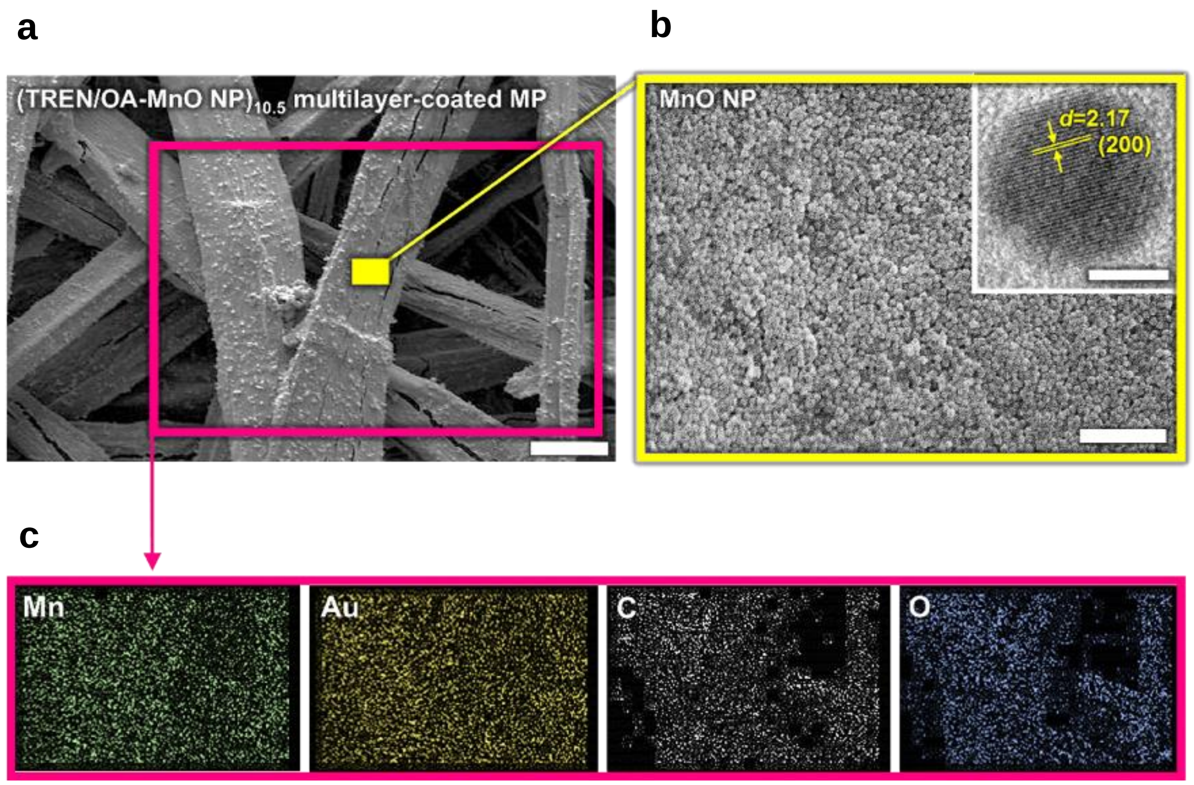

Supplementary Figure 12. Elemental mapping of MP-SC electrode. a, FE-SEM, **b,** magnified FE-SEM and HR-TEM and **c,** EDX images of (TREN/OA-MnO NP)$_{10.5}$ multilayer-coated MP electrode. In this case, MnO NPs were densely coated up to the central inside of porous MP without any agglomeration. The inset of **b** indicates the high-resolution TEM image of 11 nm-sized MnO NP with lattice fringe spacing of 2.17 Å. EDX mapping in **c** also showed a homogeneous distribution of MnO NP onto the porous MP. Scale bars in **a**, **b,** and **inset** correspond to 20 μm, 200 nm, and 5 nm, respectively.

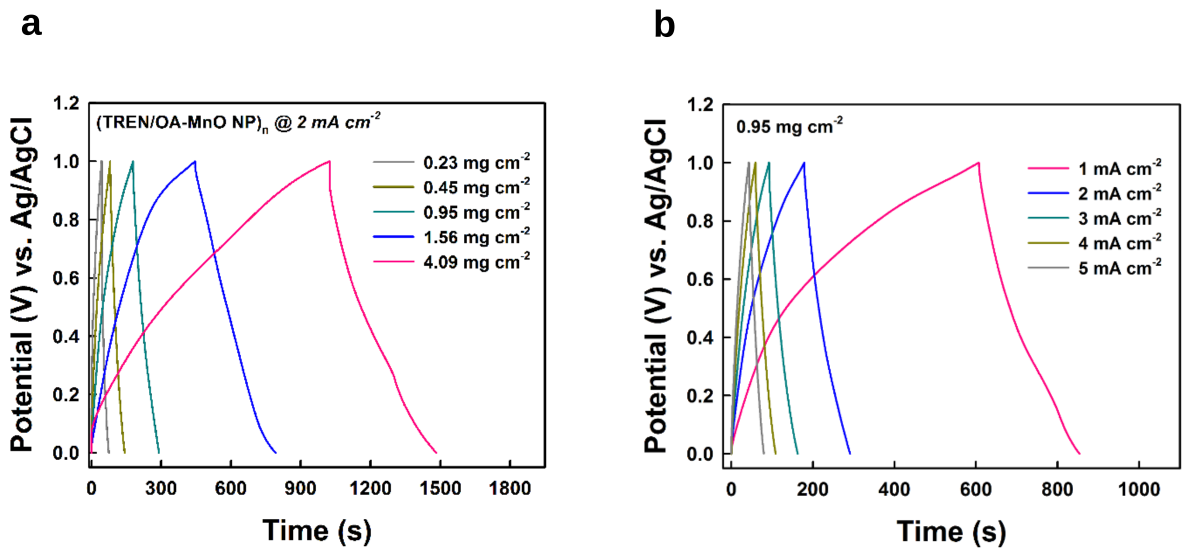

Supplementary Figure 13. Galvanostatic charge/discharge curves of (TREN/OA-MnO NP)$_n$ multilayer-coated MP-SC electrodes a, with the different mass loading of MnO NP at 2 mA·cm^{-2} and **b,** current density at the mass density of 0.95 mg·cm^{-2}.

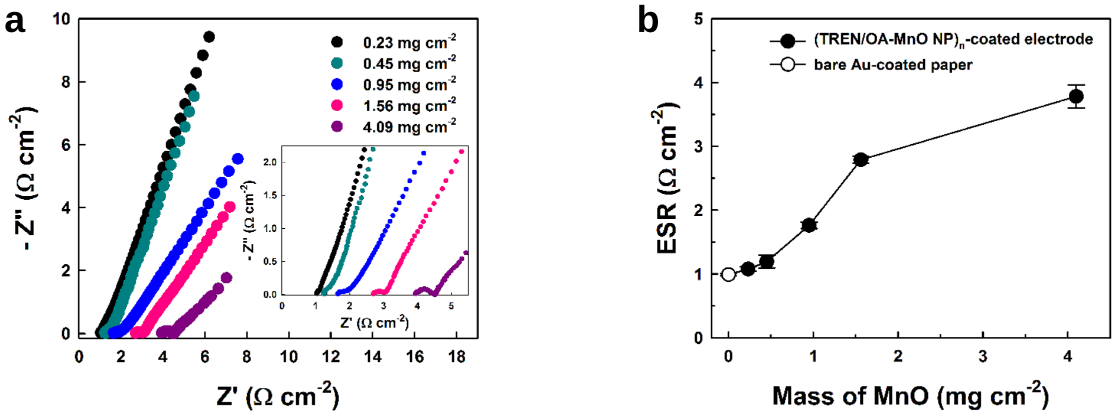

Supplementary Figure 14. Impedance studies of MP-SC electrodes. a, Nyquist plots of the (TREN/OA-MnO NP)$_n$ multilayer-coated MP-SC electrodes as a function of the mass loading of MnO NPs. **b,** ESR changes of the (TREN/OA-MnO NP)$_n$ multilayer-coated MP-SC electrodes with different mass densities of MnO NPs obtained from the Nyquist plots. The impedance spectra of the (TREN/OA-MnO NP)$_n$ multilayer electrodes shown in Supplementary Fig. 14a display two major regions of (1) the ESR and R_{ct} values at the high-frequency region and (2) a nearly straight line at the low-frequency region. In this case, the ESR (intercept at the Z-axis), the sum of the electrode and electrolyte resistance, and the R_{ct} (diameter of the semicircle) gradually increased with increasing mass loading of the MnO NPs. After increasing the mass density of the MnO NPs from 0.23 to 4.09 mg·cm^{-2}, the ESR values were measured to be 1.1, 1.2, 1.8, 2.8, and 3.9 Ω·cm^{-2}; their R_{ct} values corresponded to 0.08, 0.12, 0.15, 0.32, and 0.57 Ω·cm^{-2}, respectively. This trend was mainly due to the increase in the total mass of the MnO NPs, which interfered with the easy charge transfer[1]. In contrast, the nonporous SC electrodes exhibited significantly larger ESR and R_{ct} values than that of the MP electrodes. Fig. 3d in the main text, shown in the main text, clearly displays the different kinetics between the MP-SC and nonporous SC electrodes. In this case, the ESR values of the MP and nonporous substrate-based electrodes were measured to be 1.1 and 4.1 Ω·cm^{-2}, respectively (the inset of Fig. 3d). Notably, the diameter of the semicircular arc corresponding to R_{ct} of the nonporous electrode was substantially larger (23.4 Ω·cm^{-2}) than that of the MP-based electrode (0.08 Ω·cm^{-2}), indicating that the dense and thick MnO NP film on the nonporous substrate obstructed the charge communication between the charge carriers and NPs. The large diffusion-controlled region, the so-called 'Warburg region,' at low frequencies also demonstrates the low diffusion characteristics of the nonporous substrate-based electrode[2].

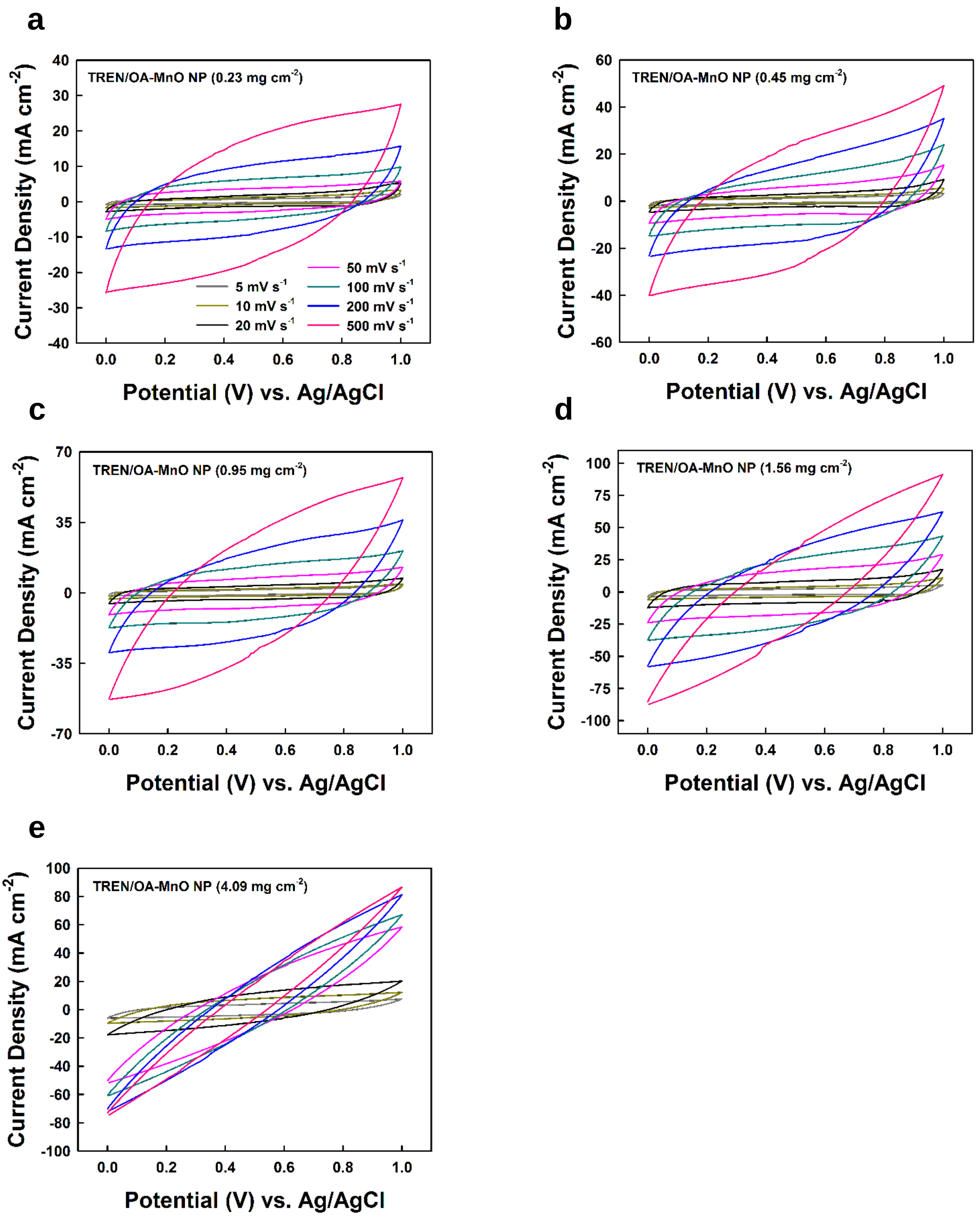

Supplementary Figure 15. CV responses of MP-based (TREN/OA-MnO NP)$_n$ electrodes with different mass density. **a,** 0.23, **b,** 0.45, **c,** 0.95, **d,** 1.56, and **e,** 4.09 mg·cm^{-2} as the scan rate increased from 5 to 500 mV·s^{-1}, respectively.

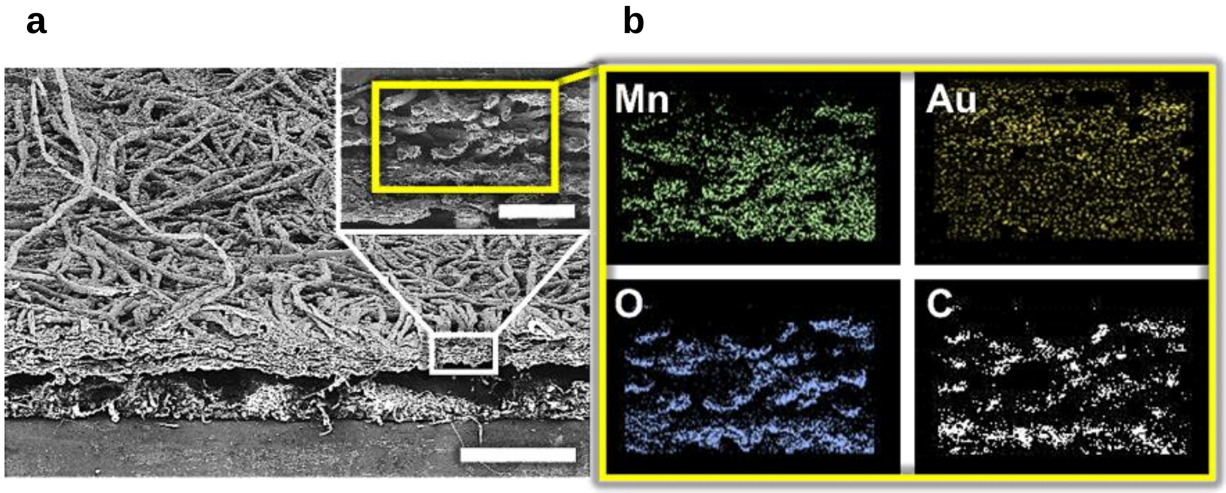

Supplementary Figure 16. **Elemental mapping of the alternating MnO/Au NP electrode.** **a,** Tilted, Cross-sectional (inset) FE-SEM images and **b,** EDX elemental mapping images of the alternating MnO/Au NP electrode. The size of scale bar in **a** and **inset** correspond to 500 μm and 50 μm, respectively.

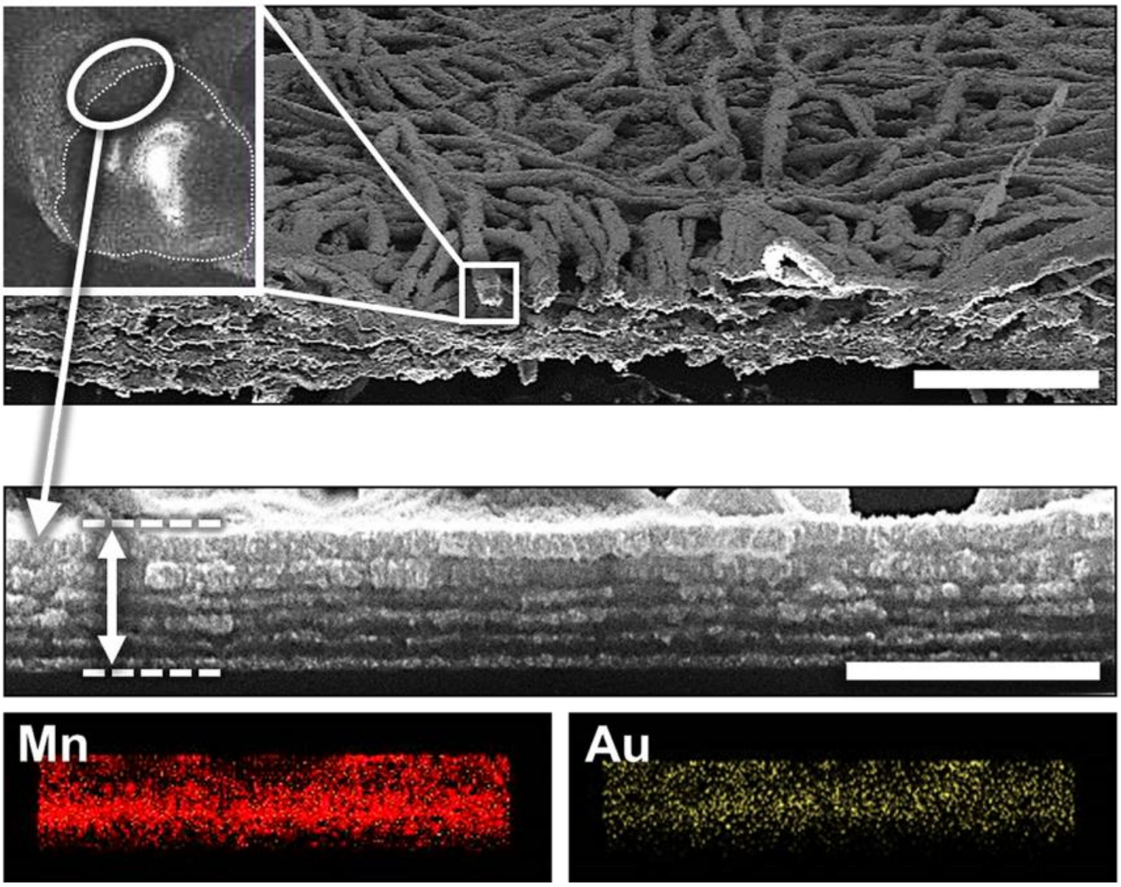

Supplementary Figure 17. FE-SEM and EDX mapping images of the alternating MnO/Au NP electrode onto the cellulose fiber. In this case, the inserted Au NPs are homogeneously distributed throughout the electrode. Scale bars in tilted (top) and cross-sectional (middle) FE-SEM images indicate 300 μm and 500 nm, respectively.

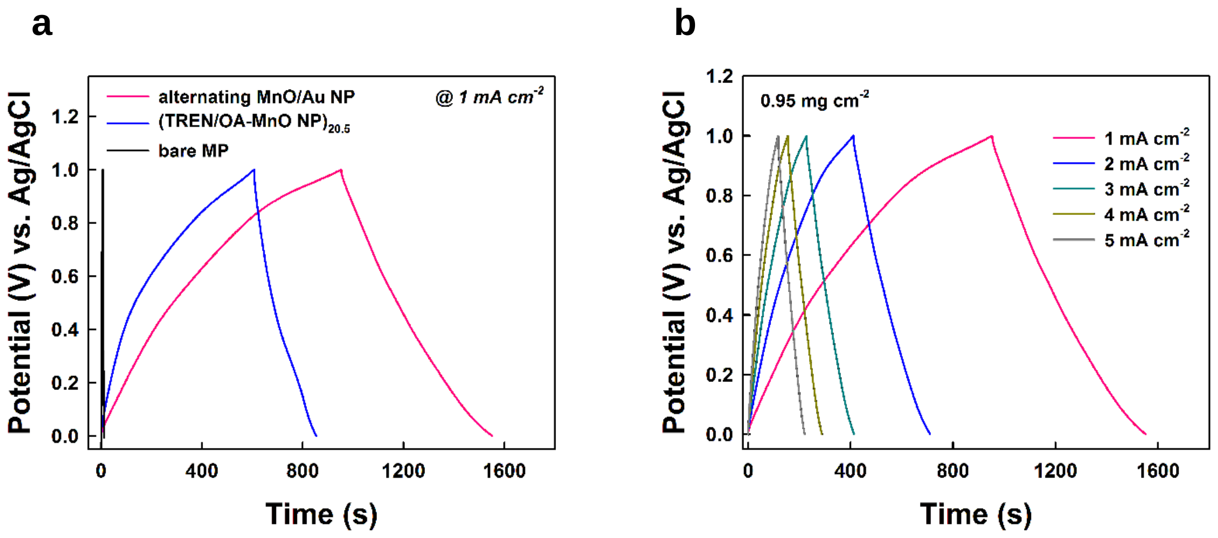

Supplementary Figure 18. Galvanostatic charge/discharge behavior of the MP-based electrodes. a, Galvanostatic charge/discharge curves of the alternating MnO/Au NP, the $(TREN/OA\text{-}MnO\ NP)_{20.5}$, and the bare MP electrodes at a current density of 1 mA·cm^{-2}, respectively. In this case, the IR drop of the alternating MnO/Au NP electrode (0.0059 V) was substantially lower than that of the $(TREN/OA\text{-}MnO\ NP)_{20.5}$ electrode (0.0142 V). **b,** Galvanostatic charge/discharge profiles of the alternating MnO/Au NP electrode (0.95 mg·cm^{-2}) with different current density of 1 – 5 mA·cm^{-2}.

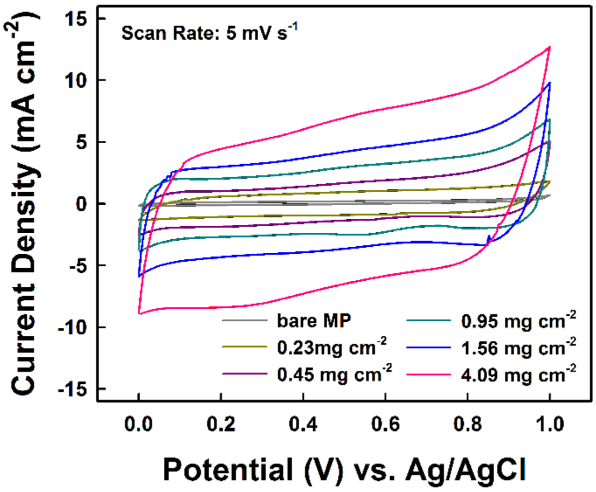

Supplementary Figure 19. CV response of the alternating MnO/Au NP electrode. Cyclic voltammograms of the alternating MnO/Au NP electrodes with different mass density at a scan rate of 5 mV·s^{-1}.

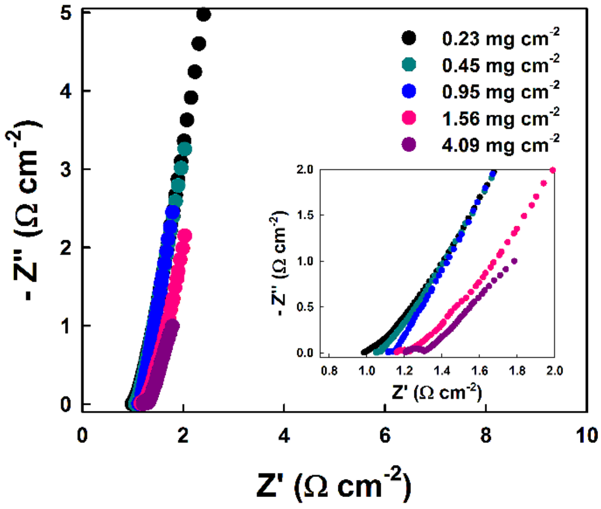

Supplementary Figure 20. EIS of the alternating MnO/Au NP electrodes. Nyquist plot of the alternating MnO/Au NP electrodes with increasing the mass density from 0.23 to 4.09 mg·cm^{-2}, respectively. The inset indicates the high frequency region of spectra.

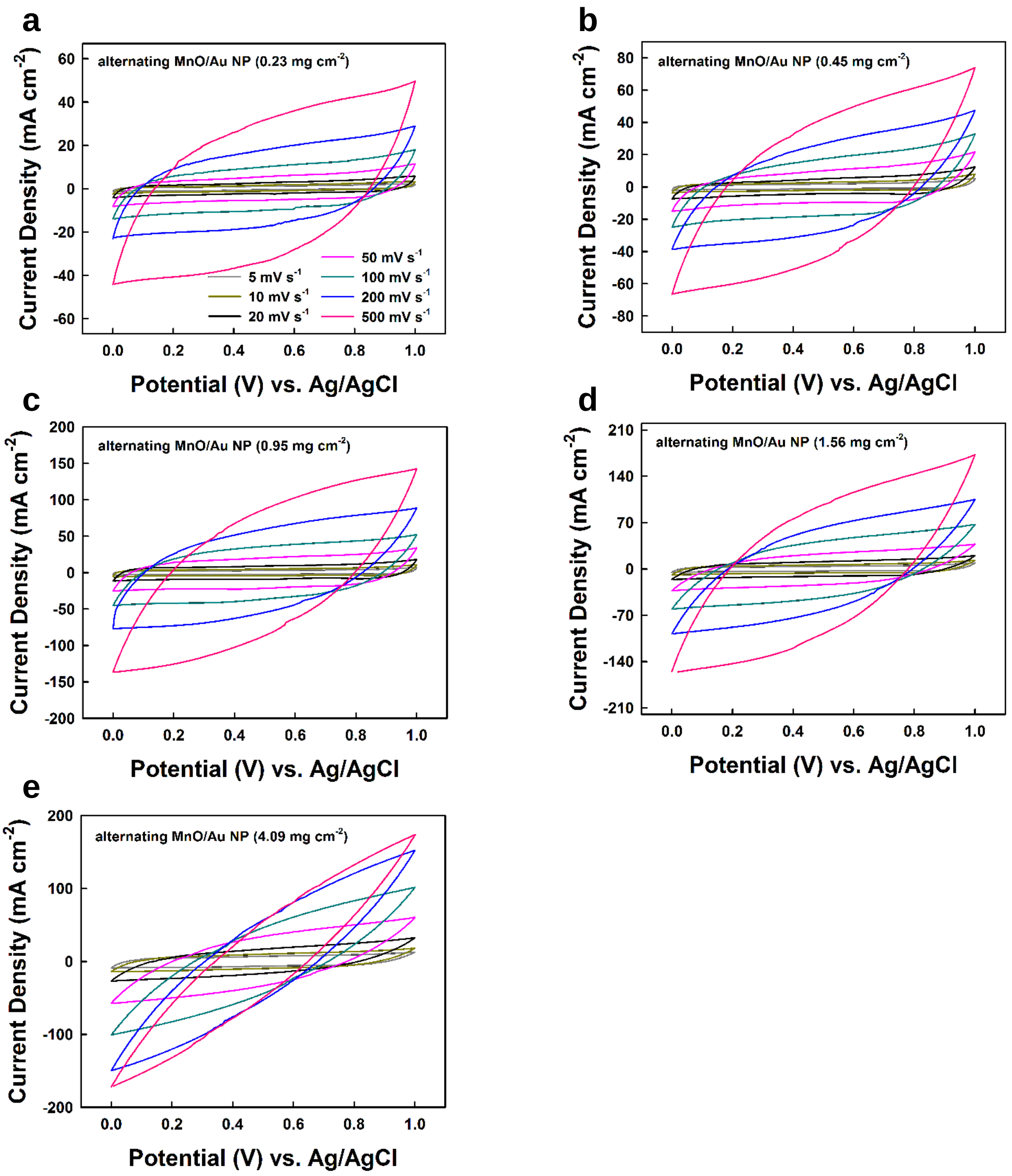

Supplementary Figure 21. CV responses of the alternating MnO/Au NP electrodes. CV scans of the alternating MnO/Au NP electrodes with different mass densities of **a,** 0.23, **b,** 0.45, **c,** 0.95, **d,** 1.56, and **e,** 4.09 mg·cm^{-2} at a scan rate of 5 – 500 mV·s^{-1}, respectively. In this case, the SC responses of the alternating MnO/Au NP electrodes show the larger and less distorted than those of $(TREN/OA\text{-}MnO\ NP)_n$ electrodes shown in Supplementary Fig. 15, demonstrating the better charge transport behavior.

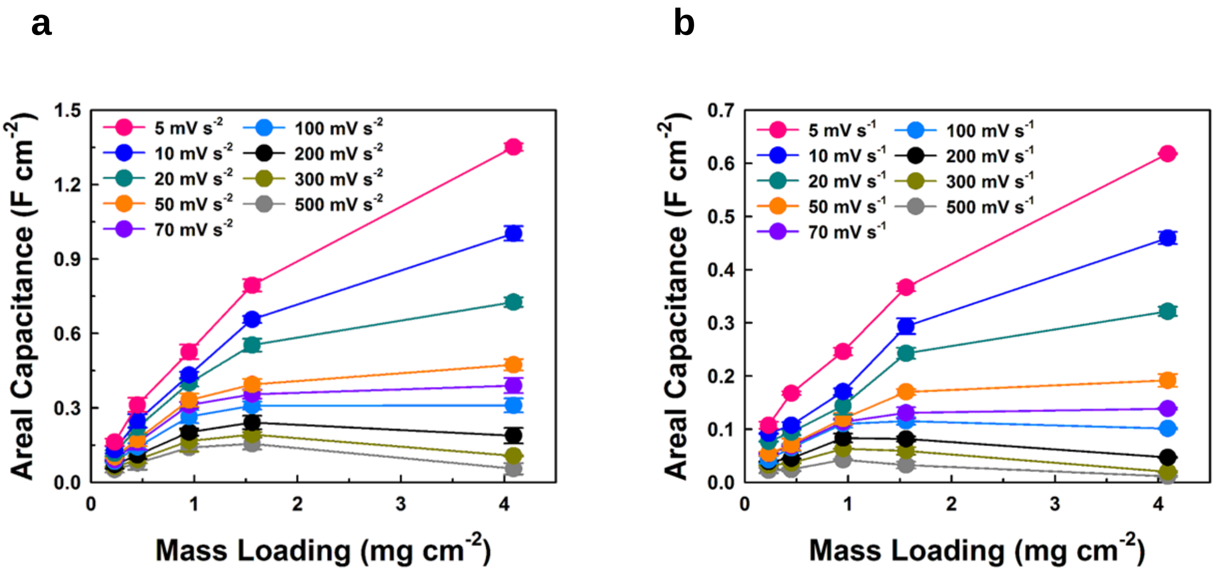

Supplementary Figure 22. Mass dependent electrochemical performance of (TREN/OA-MnO NP)$_n$-coated MP-SC electrodes. Mass dependent areal capacitance of **a,** the alternating MnO/Au NP electrodes and **b,** (TREN/OA-MnO NP)$_n$ electrode with various scan rate of 5 to 500 mV·s^{-1}. In the case of the alternating MnO/Au NP electrodes, by increasing the mass loading, the areal capacitance gradually increased until the fast scan rate of 500 mV·s^{-1} even at the mass loading of 1.56 mg·cm^{-2} (electrode area is 1 cm × 2 cm). Although the slight performance fading is observed for high mass loading of 4.09 mg·cm^{-2} from a scan rate of 200 mV·s^{-1}, these rate performance is superior to that of the (TREN/OA-MnO NP)$_n$-coated MP electrode without additional Au NP layers, indicating much better charge transport behavior.

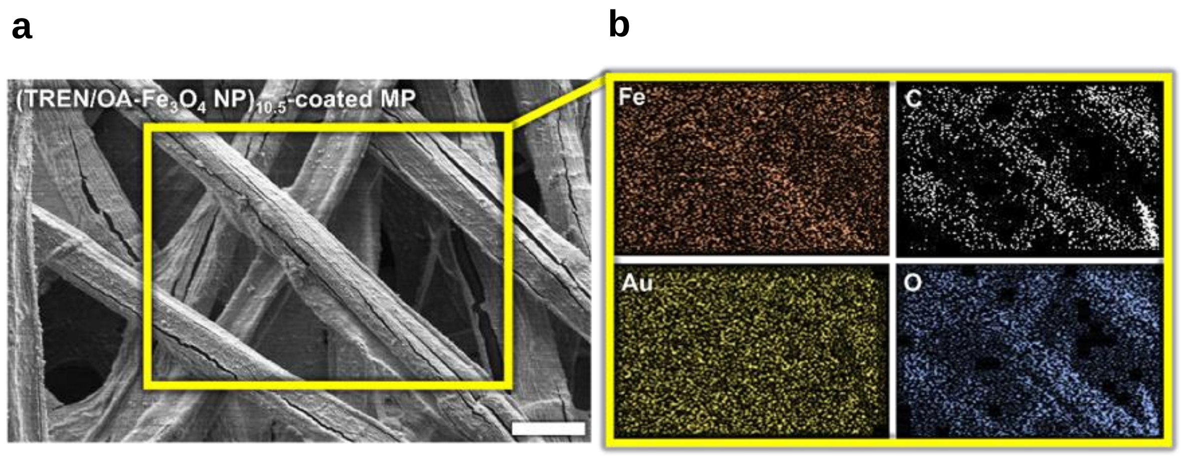

Supplementary Figure 23. Elemental mapping of the (TREN/OA-Fe_3O_4 NP)$_{10.5}$-coated MP electrode. a, FE-SEM and **b,** EDX images (yellow square). Scale bar indicates 20 μm.

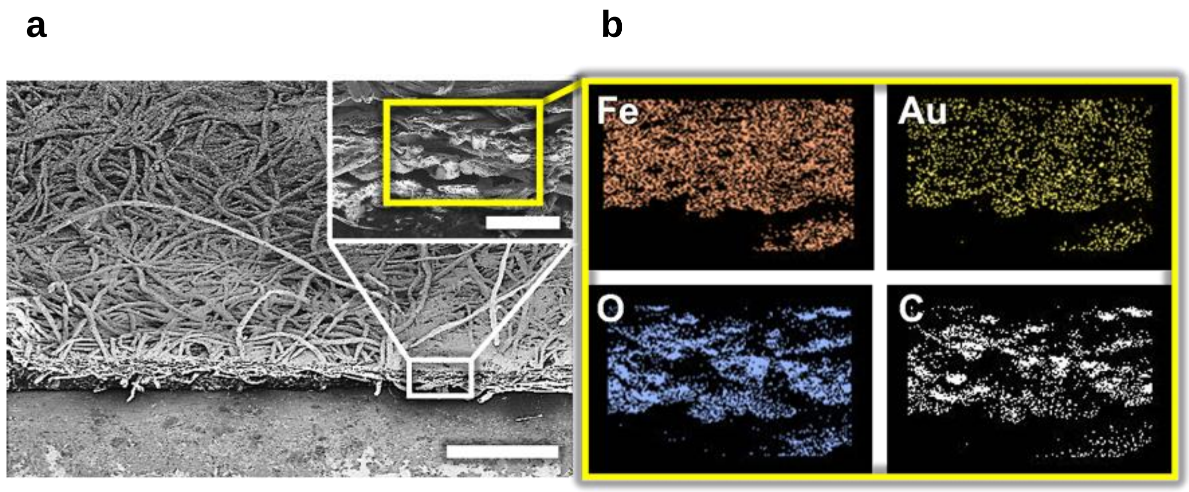

Supplementary Figure 24. **Elemental mapping of the alternating Fe_3O_4/Au NP electrode .a,** Cross-sectional FE-SEM image and **b,** EDX elemental mapping images of the alternating Fe_3O_4/Au NP electrode. The size of scale bars in FE-SEM image correspond to 500 (tilt) and 50 μm (inset), respectively.

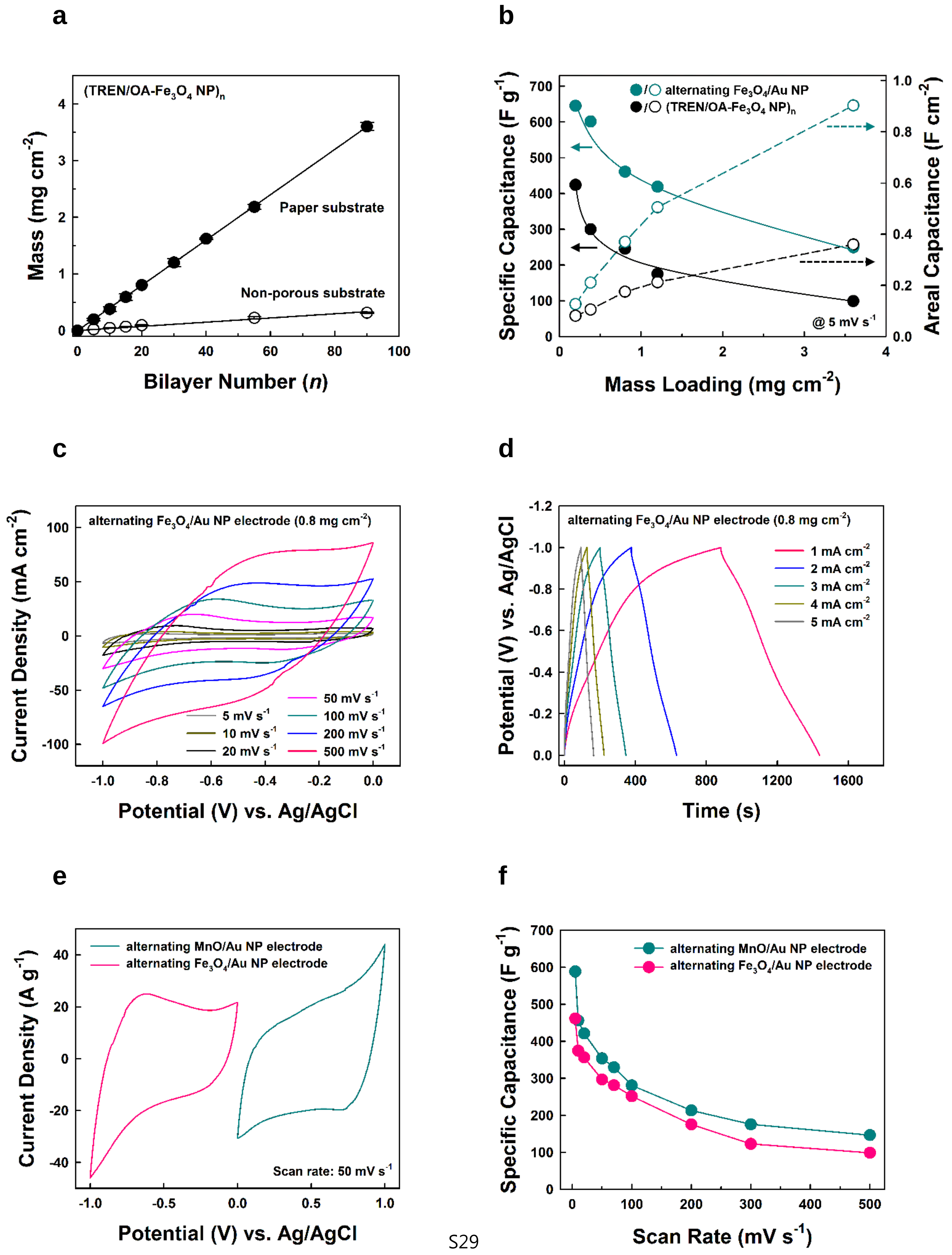

a
(TREN/OA-Fe3O4 NP)n
Mass (mg cm-2)
Paper substrate
Non-porous substrate
Bilayer Number (n)
b
alternating Fe3O4/Au NP
(TREN/OA-Fe3O4 NP)n
Specific Capacitance (F g-1)
Areal Capacitance (F cm-2)
@ 5 mV s-1
Mass Loading (mg cm-2)
c
alternating Fe3O4/Au NP electrode (0.8 mg cm-2)
Current Density (mA cm-2)
5 mV s-1
10 mV s-1
20 mV s-1
50 mV s-1
100 mV s-1
200 mV s-1
500 mV s-1
Potential (V) vs. Ag/AgCl
d
alternating Fe3O4/Au NP electrode (0.8 mg cm-2)
Potential (V) vs. Ag/AgCl
1 mA cm-2
2 mA cm-2
3 mA cm-2
4 mA cm-2
5 mA cm-2
Time (s)
e
alternating MnO/Au NP electrode
alternating Fe3O4/Au NP electrode
Current Density (A g-1)
Scan rate: 50 mV s-1
Potential (V) vs. Ag/AgCl
f
alternating MnO/Au NP electrode
alternating Fe3O4/Au NP electrode
Specific Capacitance (F g-1)
Scan Rate (mV s-1)

Supplementary Figure 25. Electrochemical properties of the (TREN/OA-Fe_3O_4 NP)$_n$-coated MP-SCs. a, Mass change of Fe_3O_4 NP deposited onto the MP substrate and the nonporous QCM electrode as a function of the bilayer number (*n*), respectively. **b,** Specific and areal capacitance of the alternating Fe_3O_4/Au NP and the (TREN/OA-Fe_3O_4 NP)$_n$ multilayer electrodes with the different mass loading of Fe_3O_4 NPs, respectively. **c,** CV responses of the alternating Fe_3O_4/Au NP electrodes (0.8 mg·cm^{-2}) as a function of the scan rate. **d,** Galvanostatic charge/discharge curves of the alternating Fe_3O_4/Au NP electrodes at the current density ranging from 1 to 5 mA·cm^{-2}. **e,** CVs of the alternating MnO/Au NP electrode and the alternating Fe_3O_4/Au NP electrode recorded at a scan rate of 50 mV·s^{-1}. **f,** Specific capacitance of the alternating MnO/Au NP electrode and the alternating Fe_3O_4/Au NP electrode as a function of the scan rate (5 to 500 mV·s^{-1}).

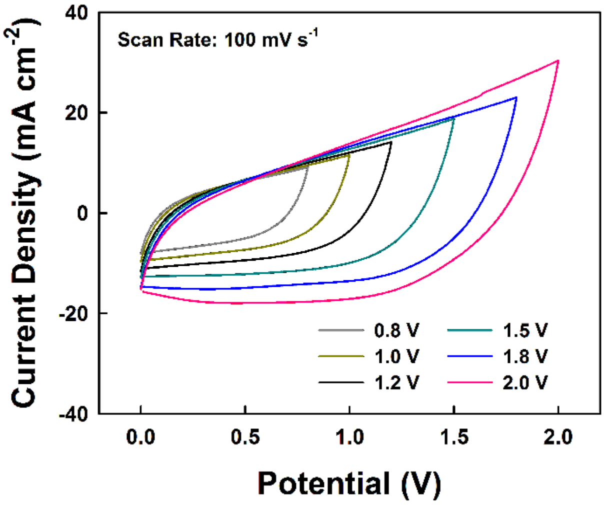

Supplementary Figure 26. Potential dependent CVs of solid-state MP-ASC. CVs of solid-state MP-ASC at a scan rate of 100 mV·s^{-1} with increasing working voltage from 0 to 2 V.

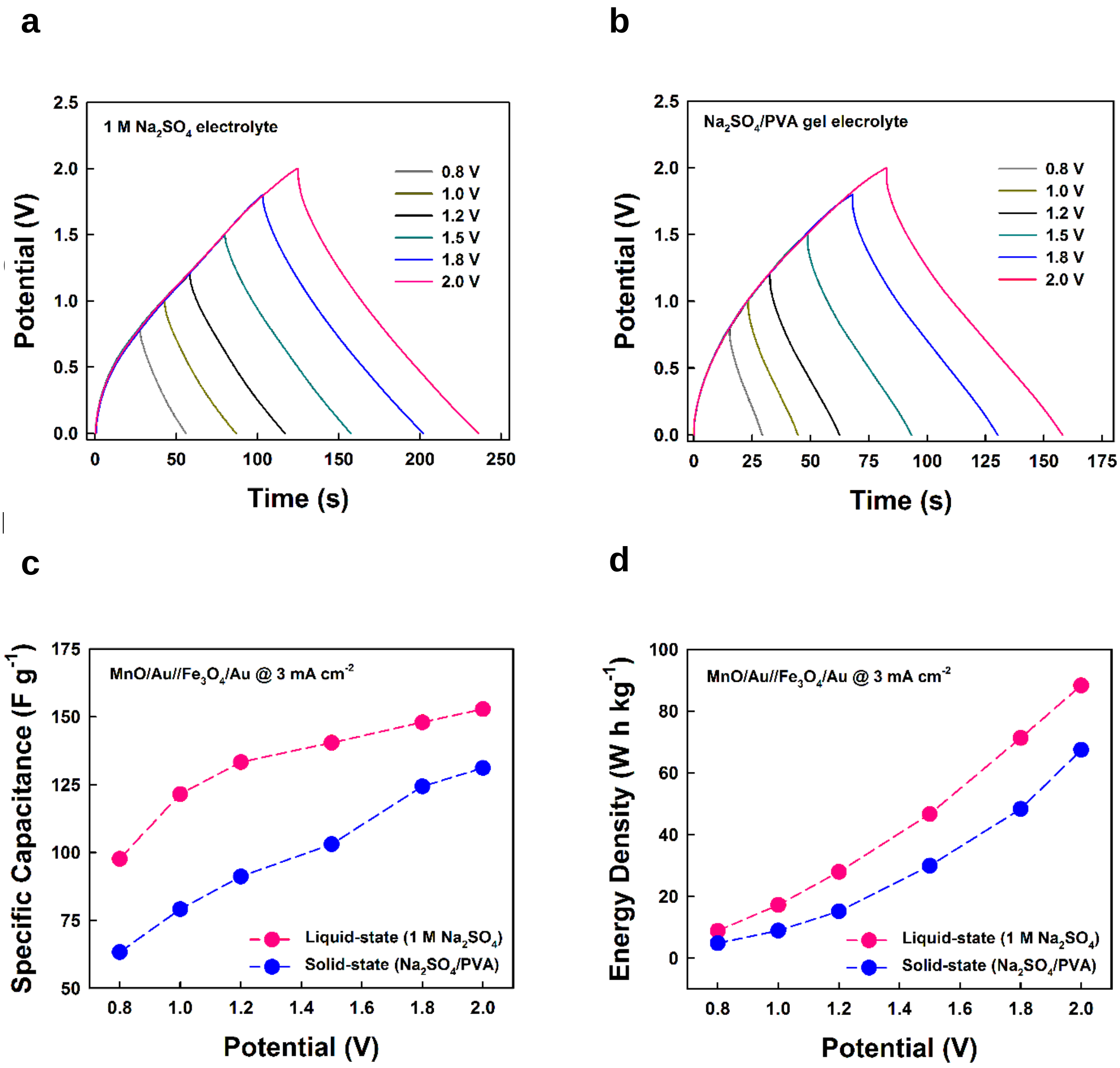

Supplementary Figure 27. Electrochemical characterization of liquid-state and solid-state ASCs. Galvanostatic charge/discharge curves of **a,** liquid-state and **b,** solid-state MP-ASC at a current density of 3 mA·cm^{-2} with increasing the potential window up to 2 V. **c,** Trace of the specific capacitance of the MP-ASC with different working voltage. These values were obtained from the discharge profile in Fig. S22a. **d,** Change in energy density of the MP-ASC according to voltage window. In this case, the specific capacitance of the liquid- and the solid-state MP-ASC gradually increases with increasing the potential window from 0.8 to 2 V. These results demonstrate that the energy capability can be improved by more than ten times according to the equation of $E = 1/2CV^2$ (see methods).

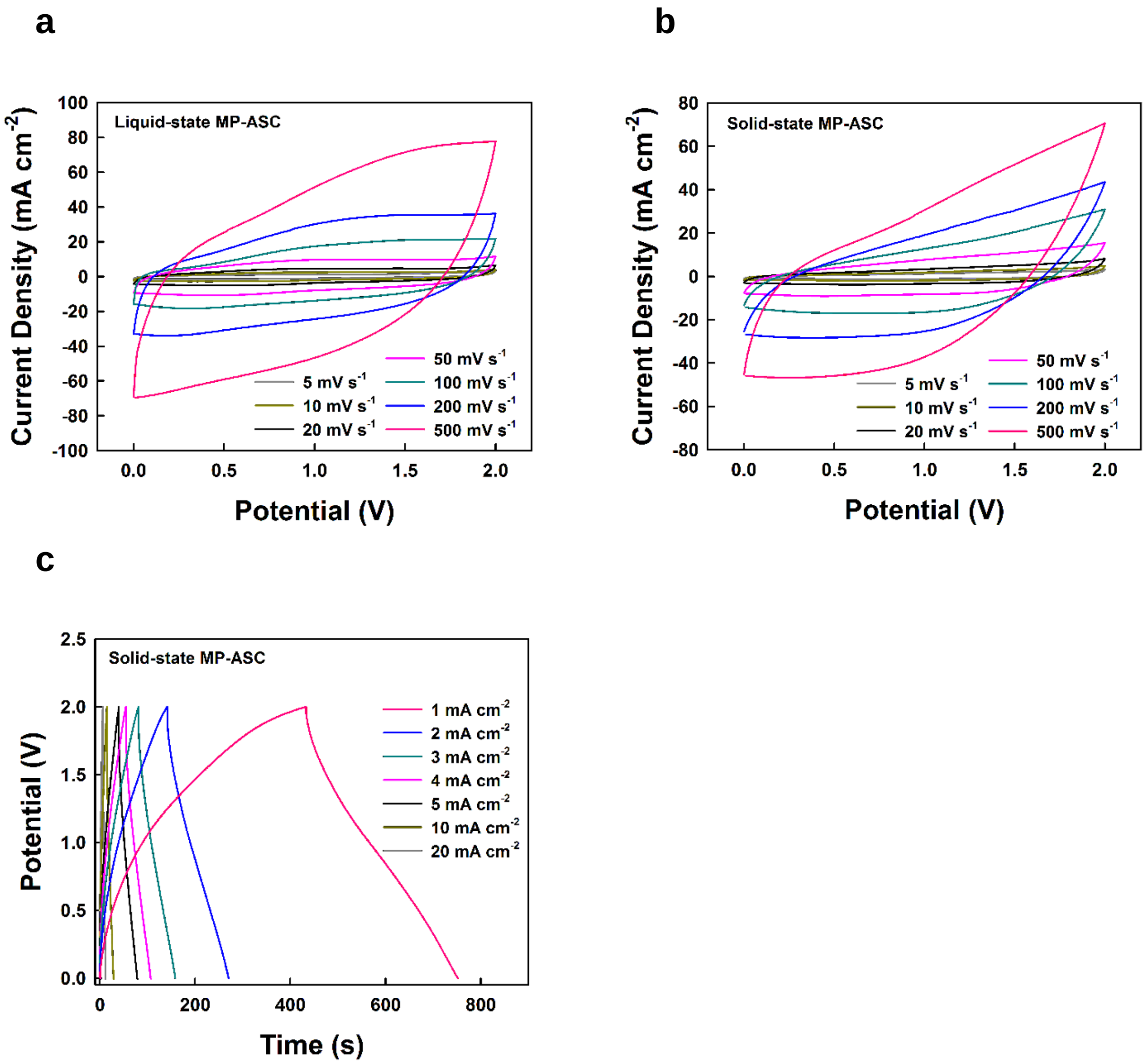

Supplementary Figure 28. Electrochemical properties of MP-ASCs. CVs of **a,** the liquid and **b,** solid-state MP-ASC at the different scan rates ranging from 5 to 500 $mV \cdot s^{-1}$. **c,** Galvanostatic charge/discharge curves of the solid-state MP-ASC with different current density.

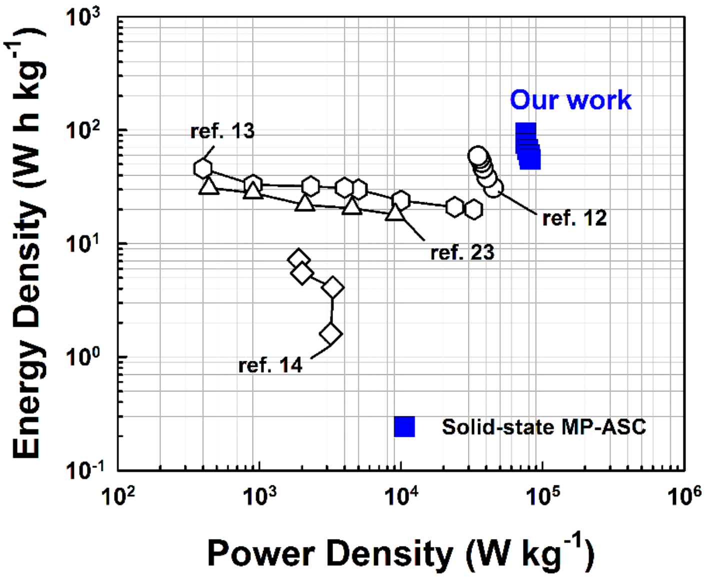

Supplementary Figure 29. Ragone plots of the solid-state MP-ASCs. Performance comparison between the solid-state MP-ASC (in our study) and the previously reported ASC based on total mass of active energy materials.

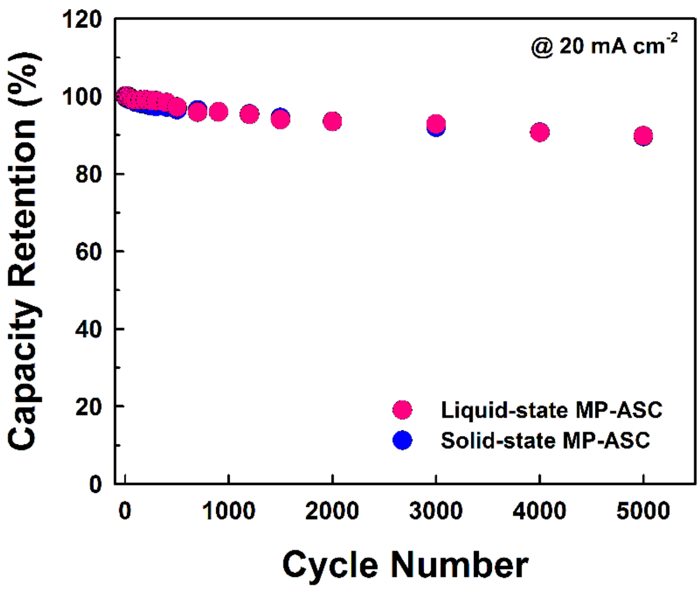

Supplementary Figure 30. Cycling retention of the solid-state and liquid-state ASCs. All data points collected from galvanostatic charge/discharge measurement at a current density of 20 mA·cm^{-2}.

Supplementary Table 1. Performance comparison of textile-based supercapacitor electrodes (three-electrode configuration)

Active material	Substrate	Specific capacitance ($F·g^{−1}$) (mass loading of active materials)	Areal capacitance ($F·cm^{−2}$)	Ref.
MnO/Au	Cellulose paper	709 (0.23 $mg·cm^{−2}$ at 5 $mV·s^{−1}$)	1.35 (4.09 $mg·cm^{−2}$ at 5 $mV·s^{−1}$)	**Our work**
MnO	Cellulose paper	481 (0.23 $mg·cm^{−2}$ at 5 $mV·s^{−1}$)	0.617 (4.09 $mg·cm^{−2}$ at 5 $mV·s^{−1}$)	**Our work**
MnO_2/CNT	Polyester fiber	410 (0.06 $mg·cm^{−2}$ at 5 $mV·s^{−1}$)	~0.54 (3.8 $mg·cm^{−2}$ at 5 $mV·s^{−1}$)	3
MnO_2	Carbon fabric	197.4 (0.76 $mg·cm^{−2}$ at 1.3 $A·g^{−1}$)	0.15 (0.76 $mg·cm^{−2}$ at 1 $mA·cm^{−2}$)	4
MnO_2	Carbon cloth	425 (0.54 $mg·cm^{-2}$ at 10 $mV·s^{-1}$)	0.23 (0.54 $mg·cm^{−2}$ at 0.13$mA·cm^{−1}$)	5
MnO_2 NR*/CNP*	Carbon fabric	800 (0.201 $mg·cm^{−2}$ at 5 $mV·s^{−1}$)	0.109 (0.562 $mg·cm^{−2}$ at 5 $mV·s^{−1}$)	6
MONRAs*	Carbon fabric	~505 (~0.07 $mg·cm^{−2}$ at 2 $mV·s^{−1}$)	–	7
WO_{3-x}@Au@MnO_2	Carbon fabric	588 (0.088 $mg·cm^{−2}$ at 10 $mV·s^{−1}$)	–	8
MnO_2/graphene	Polyester fabric	332 (0.345 $mg·cm^{−2}$ at 2 $mV·s^{−1}$)	0.079 (0.345 $mg·cm^{−2}$ at 2 $mV·s^{−1}$)**	9
MnO_2/Zn_2SnO_4	Carbon fiber	621.6 (at 2 $mV·s^{−1}$)	–	10

* NR: nanorod, CNP: carbon nanoparticle, MONRAs: manganese dioxide nanorod arrays.

** Calculated from given data in the literature

Supplementary Table 2. Performance comparison of flexible SCs in two-electrode measurement

Positive electrode (cathode)	Negative electrode (anode)	Electrolyte	Potential (V)	Mass (active material)	Specific capacitance ($F\cdot g^{-1}$)	Power ($kW\cdot kg^{-1}$) max.	Energy ($W\cdot h\cdot kg^{-1}$) max.	Ref.
**Textile substrate (polyester, cotton, carbon fiber, carbon fabric paper etc.)**								
MnO/Au	Fe_3O_4/Au	Na_2SO_4 (1 M)	0 − 2	3.1 mg	222 (1 $mA\cdot cm^{-2}$)	128.9	121.5	**Our work**
		Na_2SO_4/PVA	0 − 2	3.1 mg	176.1 (1 $mA\cdot cm^{-2}$)	81.7	95.1	**Our work**
MnO_2-CNT	rMnO_2-CNT	Na_2SO_4 (0.5 M)	0 − 1	~1.6 mg	−	~13	~17	3
graphene/MnO_2	CNT	Na_2SO_4 (0.5M)	0 − 1.5	−	−	110	12.5	11
MnO_2/ SWNT	In_2O_3/ SWNT	Na_2SO_4 (1 M)	0 − 2	0.54 mg	184	~270	25.5	12
GF*/CNT/MnO_2	GF/CNT/PPy*	Na_2SO_4 (0.5 M)	0 − 1.6	−	−	10.3	22.2	13
H-TiO_2*@ MnO_2	H-TiO_2@ C	LiCl/PVA	0 − 1.8	~0.23 $mg\cdot cm^{-2}$	139.6 (1.1 $A\cdot g^{-1}$)	45	59	14
MnO_2/CNT	Fe_2O_3/CNT	Na_2SO_4/PVA	0 − 2	0.33 mg	82.4 (0.1 $A\cdot g^{-1}$)	32.7	45.8	15
MnO_2/CNT	MnO_2/CNT	H_2SO_4/PVA	0 − 0.8	−	324 (0.5 $A\cdot g^{-1}$)	3.3	7.2	16

RuO_2-IL-CMG*	IL-CMG	H_2SO_4/PVA	0 − 1.8	–	175 (0.5 A·g^{-1})	6.8	19.7	17
CoO@NiO/ACT*	ACT/graphene	PVA-KOH	0 − 1.6	–	147.6 (10 mA·cm^{-2})	9.53	52.26	18
$NiCo_2O_4$@$NiCo_2O_4$/ ACT	ACT	PVA-KOH	0 − 1.6	2.63 mg·cm^{-2}	179 (1 mA·cm^{-2})	8.4	83.6	19
$NiCo_2O_4$/CC*	PGP*	PVA-LiOH	0 − 1.8	~ 2.2 mg	71.32	11.36	60.9	20
**Other flexible substrate (metal, metal foam, sponge, metal-coated plastic etc.)**								
graphene/MnO_2	ACN*	Na_2SO_4 (1 M)	0 − 1.8	–	113.5 (1 mV·s^{-1})	198	51.1	21
MnO_2-NPG*	PPy-NPG	$LiClO_4$ (1 M)	0 − 1.8	0.11 mg	193 (2 A·g^{-1})	25	86	22
NPG/ MnO_2	NPG/ MnO_2	Li_2SO_4 (2 M)	0 − 0.8	-	~1,145 (50 mV·s^{-1})	~16	~57	23
aMEGO*/MnO_2	aMEGO	Na_2SO_4 (1 M)	0 − 2	2.1 mg	175 (0.25 A·g^{-1})	32.3	24.3	24
MnO_2-graphene	CNT-graphene	KCl/PAAK*	0 − 1.8	-	69.4 (0.5 A·g^{-1})	~9.2	31.8	25

* GF: graphene foam, PPy: polypyrrole, H-TiO_2: hydrogen-treated TiO_2, IL-CMG: ionic liquid functionalized-chemically modified graphene, ACT: activated carbon textile, CC: carbon cloth, PGP: porous graphene paper, ACN: activated carbon nanofiber, aMEGO: activated microwave expanded graphene oxide, NPG: nanoporous gold, CCgaphene: chemically converted graphene, PAAK: potassium polyacrylate

Supplementary Table 3. Comparison of the areal power and energy densities of the flexible SCs in two-electrode system.

**Active material**	**Mass (active material)**	**Electrolyte**	**Areal power ($mW \cdot cm^{-2}$) max.**	**Areal energy ($\mu W \cdot h \cdot cm^{-2}$) max.**	**Ref.**
MnO/Au//Fe_3O_4/Au	3.1 mg	Na_2SO_4 (1 M)	15.1	267.3	**Our work**
	3.1 mg	Na_2SO_4/PVA	9.5	174.7	**Our work**
RGO*/MnO_2//RGO	15 mg	Na_2SO_4 (1 M)	3.8	35.1	26.
MnO_2/CNT (symmetric configuration)	31.6 mg	Na_2SO_4 (1 M)	2.5	46	27
GO/PPy (symmetric configuration)	2.04 mg	LiCl/PVA gel	4	10.8	28
RGO/carbon paper (symmetric configuration)	30.71 mg	H_2SO_4/PVA gel	20	50	29
CDC*/TiC//AC*	-	H_2SO_4 (1 M)	31	1.6	30

* RGO: reduced graphene oxide, CDC: carbide-derived carbon, AC: activated carbon

Supplementary References

1. Wan, C., Yuan, L. & Shen, H. Effects of electrode mass-loading on the electrochemical properties of porous MnO_2 for electrochemical supercapacitor. *Int. J. Electrochem. Sci.* **9**, 4024−4038 (2014).
2. Stoller, M. D., Park, S., Zhu, Y., An, J. & Ruoff., R. S. Graphene-based ultracapacitors, *Nano Lett.* **8**, 3498−3502 (2008).
3. Hu, L. *et al.* Symmetric MnO_2-carbon nanotube-textile nanostructures for wearable pseudocapacitors with high mass loading. *ACS Nano* **5**, 8904−8913 (2011).
4. Yang, P. *et al.* Low-cost high-performance solid-state asymmetric supercapacitors based on MnO_2 nanowires and Fe_2O_3 nanotubes, *Nano Lett.* **14**, 731−736 (2014).
5. Chen, Y.-C. *et al.* Highly flexible supercapacitors with manganese oxide nanosheet/carbon cloth electrode. *Electrochim. Acta* **56**, 7124 (2011).
6. Yuan, L. *et al.* Flexible solid-state supercapacitors based on carbon nanoparticles/MnO_2 nanorods hybrid structure. *ACS Nano* **6**, 656−661 (2012).
7. Yu, M. *et al.* Manganese dioxide nanorod arrays on carbon fabric for flexible solid-state supercapacitors. *J. Power Source* **239**, 64−71 (2013).
8. Lu, X. *et al.* WO_{3-x}@Au@MnO_2 core-shell nanowires on carbon fabric for high-performance flexible supercapacitors. *Adv. Mater.* **24**, 938−944 (2012).
9. Guo, M.-X., Bian, S.-W., Shao, F., Liu, S. & Peng, Y.-H. Hydrothermal synthesis and electrochemical performance of MnO_2/graphene/polyester composite electrode materials for flexible supercapacitors. *Electrochim. Acta* **209**, 486−497 (2016).
10. Bao, L., Zang, J. & Li, X. Flexible Zn_2SnO_4/MnO_2 core/shell nanocable-carbon microfiber hybrid composites for high-performance supercapacitor electrodes. *Nano Lett.* **11**, 1215−1220 (2011).
11. Yu, G. *et al.* Solution-processed graphene/MnO_2 nanostructured textiles for high-performance electrochemical capacitors. *Nano Lett.* **11**, 2905−2911 (2011).
12. Chen, P.-C., Shen, G., Shi, Y., Chen, H. & Zhou, C. Preparation and characterization of flexible asymmetric supercapacitors based on transition-metal-oxide nanowire/single-walled carbon nanotube hybrid thin-film electrodes. *ACS Nano* **4**, 4403−4411 (2010).
13. Liu, J. *et al.* High-performance flexible asymmetric supercapacitors based on a new graphene foam/carbon nanotube hybrid film. *Energy Environ. Sci.* **7**, 3709−3719 (2014).
14. Lu, X. *et al.* H-TiO_2@MnO_2//H-TiO_2@C core-shell nanowires for high performance and flexible asymmetric supercapacitors. *Adv. Mater.* **25**, 267−272 (2013).
15. Gu, T. & Wei, B. High-performance all-solid-state asymmetric stretchable supercapacitors based on wrinkled MnO_2/CNT and Fe_2O_3/CNT macrofilms. *J. Mater. Chem. A* **4**, 12289 (2016).
16. Ko, W.-Y., Chen, Y.-F., Lu, K.-M. & Lin, K.-J. Porous honeycomb structures formed from interconnected MnO2 sheets on CNT-coated substrates for flexible all-solid-

state supercapacitors. *Sci. Rep.* **6**, 18887 (2016).

17 Choi, B. G. *et al.* High performance of a solid-state flexible asymmetric supercapacitor based on graphene films. *Nanoscale* **4**, 4983−4988 (2012).

18 Gao, Z., Song, N. & Li, X. Microstructural design of hybrid CoO@NiO and graphene nano-architectures for flexible high performance supercapacitors. *J. Mater. Chem. A* **3**, 14833−14844 (2015).

19 Gao, Z., Song, N., Zhang, Y. & Li, X. Cotton textile enable, all-solid-state flexible supercapacitors. *RSC Adv.* **5**, 15438−15447 (2015).

20 Gao, Z. *et al.* Flexible all-solid-state hierarchical $NaCo_2O_4$/porous graphene paper asymmetric supercapacitors with an exceptional combination of electrochemical properties. *Nano Energy* **13**, 306−317 (2015).

21 Fan, Z. *et al.* Asymmetric supercapacitors based on graphene/MnO_2 and activated carbon nanofiber electrodes with high power and energy density. *Adv. Funct. Mater.* **21**, 2366−2375 (2011).

22 Hou, Y. *et al.* Nanoporous metal based flexible asymmetric pseudocapacitors. *J. Mater. Chem. A* **2**, 10910 (2014).

23 Lang, X., Hirata, A., Fujita, T. & Chen, M. Nanoporous metal/oxide hybrid electrodes for electrochemical supercapacitors. *Nat. Nanotech.* **6**, 232−236 (2011).

24 Zhao, X. *et al.* Incorporation of manganese dioxide within ultraporous activated graphene for high-performance electrochemical capacitors. *ACS Nano* **6**, 5404−5412 (2012).

25 Zhang, Z. *et al.* Facile synthesis of 3D MnO_2-graphene and carbon nanotube-graphene composite networks for high-performance, flexible, all-solid-sate asymmetric supercapacitors. *Adv. Energy Mater*. **4**, 1400064 (2014).

26 Sumboja, A., Foo, C. Y., Wang, X. & Lee, P. S. Large areal mass, flexible and free-standing reduced graphene oxide/manganese dioxide paper for asymmetric supercapacitor device. *Adv. Mater*. **25**, 2809−2815 (2013).

27 Chen, H. *et al.* Oxygen evolution assisted fabrication of highly loaded carbon nanotube/MnO_2 hybrid films for high-performance flexible pseudocapacitors. *Small* **12**, 2035−2045 (2016).

28 Cao, J. *et al.* Three-dementional graphene oxide/polypyrrole composite electrodes fabricated by one-step electrodeposition for high performance supercapacitors. *J. Mater. Chem. A* **3**, 14445 (2015).

29 Zhang, L. *et al.* Steamed water engineering mechanically robust graphene films for high-performance electrochemical capacitive energy storage. *Nano Energy* **26**, 668−676 (2015).

30 Huang, P. *et al.* On-chip and freestanding elastic carbon films for micro-supercapacitors. *Science* **351**, 691−695 (2016).
